# Two decades of Landsat images reveal spatial and temporal dynamics of invasion and improve species distribution models

**DOI:** 10.1002/eap.70331

**Published:** 2026-11-04

**Authors:** Thomas A. Lake, Ryan D. Briscoe Runquist, David A. Moeller

**Affiliations:** ^1^ Department of Plant and Microbial Biology University of Minnesota St. Paul Minnesota USA; ^2^ Center for Geospatial Analytics North Carolina State University Raleigh North Carolina USA

**Keywords:** biological invasions, convolutional neural network, deep learning, Landsat satellite imagery, population dynamics, process‐based distribution models, satellite image time‐series

## Abstract

Developing accurate and cost‐effective methods to detect and predict the spread of invasive species remains an ongoing challenge. Species distribution models (SDMs) are widely used to predict invasion under current and future climates and inform management strategies. However, occurrence datasets are often spatially and temporally biased due to unstructured and opportunistic data collection. Remote sensing of individual species over broad spatial and temporal scales has become increasingly feasible with the accumulation of satellite images and the development of convolutional neural networks. In this study, we used 21 years of archival Landsat satellite imagery (2000–2020) to train a land use/land cover (LULC) classification algorithm using a temporal convolutional neural network to predict occurrence of the invasive species, leafy spurge (*Euphorbia virgata*), across Minnesota. We validated our predictions with an independent dataset. First, we show that leafy spurge has expanded from 1067 km^2^ in 2000–2002 to 7156 km^2^ in 2018–2020, a 570% increase. Drought severity modulated the predicted area invaded and was associated with fluctuations over time. Second, we tracked changes in probability over time for individual pixels and showed that invasion has been concentrated in two largely disjunct regions that differ in climate. Third, our remotely sensed predicted occurrence dataset and community science dataset were closer to roadsides, suggesting that roadside disturbance facilitates invasion. Last, we showed that SDMs built using remotely sensed occurrences had higher discrimination, were less overfit, and performed better outside of urban areas. Overall, 21 years of archival satellite imagery showed rapid expansion in two geographically distinct regions, invasion associated with roads, and improved predictions of invasion.

## INTRODUCTION

Predicting the distribution and abundance of individual species is fundamental to basic and applied problems in ecology, biogeography, and conservation (Meyer et al., 2015). However, occurrence datasets are typically spatially biased, which has compromised our ability to fairly test biogeographic predictions (e.g., abundant center hypothesis) and to accurately forecast species' range shifts (Garcia‐Rosello et al., 2023; Hughes et al., 2021). Recent developments in remote sensing have provided avenues for identifying individual species from satellite images with considerable accuracy (Randin et al., 2020; Wang & Gamon, 2019). Although rarely attempted, remote sensing models increasingly have the potential to characterize the distribution and population trends of individual species over large geographic areas and long time periods (Bradley & Mustard, 2006; He et al., 2015; Randin et al., 2020; Wang et al., 2024; Wang & Diao, 2014).

Among the most frequent and important uses of occurrence datasets is for the construction of presence‐only species distribution models (SDMs) or environmental niche models (hereafter SDMs) (Elith & Leathwick, 2009; Guisan & Zimmermann, 2000; Miller, 2010). These models are widely used to forecast range shifts with climate change and anticipate invasions (Franklin, 2023). In most models, occurrences are acquired from publicly available databases (e.g., GBIF) that accumulate records from many sources and over long time periods (Di Cecco et al., 2021; Larson et al., 2020). Although these occurrence datasets are often large, they rarely sample the landscape randomly or accurately represent changes in abundance over time (Beck et al., 2014; Di Cecco et al., 2021; Geurts et al., 2023; Meyer et al., 2016).

SDMs (presence‐only) face a few challenges. Spatial bias in occurrence datasets is problematic because it influences the distribution of occurrence‐environment relationships. In addition, SDMs assume range equilibrium but this assumption is often violated for species with rapidly changing distributions (e.g., invasions). In these species, occurrences may be concentrated in regions first colonized and poorly represented at expanding range margins (Bowler et al., 2022; Isaac & Pocock, 2015). Similarly, observations may be concentrated spatially or temporally where and when effort is greater, such as in urban centers, along roadsides, or during times of increased participation in community science (Bowler et al., 2022; Kadmon et al., 2004). When data collection biases translate to biases in environmental associations, SDMs often fail to accurately predict suitable but unoccupied areas (Briscoe Runquist et al., 2019; Lustenhouwer & Parker, 2022; Phillips et al., 2009; Yates et al., 2018). Considerable effort has been dedicated to developing methods that reduce spatial bias (Franklin, 2023). While those approaches are helpful in some cases, they typically do not eliminate bias and generating accurate forecasts of range expansion continues to be challenging (Fourcade et al., 2014; Jarnevich et al., 2015; Lake et al., 2020; Syfert et al., 2013). SDMs that use remotely sensed data may be able to help correct for the temporal and spatial variability in volunteer effort, by leveraging spectral and temporal dependencies in reflectance in order to make landscape‐level occurrence predictions.

Traditional SDMs are built based on patterns of occurrence but do not account for variation in abundance or demography. Process‐based SDMs relate biological processes (such as demographic factors) to environmental variability (Briscoe et al., 2019; Dormann et al., 2012; Higgins et al., 2020). Process‐based models may better predict habitat suitability and the extent of range shifts with climate change (Dormann et al., 2012; Eckhart et al., 2011; Van der Meersch et al., 2024). Despite their advantages, these models are rare because they require long‐term monitoring (or experimentation) across a species' range (Briscoe et al., 2019; Dormann et al., 2012).

Advances in remote sensing, satellite imagery systems, and computing have enabled detailed mapping and demographic modeling of plant populations (Battison et al., 2024; Bradley, 2014; Fenollosa et al., 2025; Müllerová et al., 2023; Olsoy et al., 2024). Multispectral imagery archives are a rapidly expanding resource that can be used to map all levels of biodiversity across the globe (Randin et al., 2020; Thapa‐Magar et al., 2025). The Landsat satellite imagery archive is unique due to its deep temporal catalogue: the global dataset includes images captured approximately every 8–16 days for over 50 years (1972–present). Shifts in distribution and abundance have been detected by comparing historical and contemporary imagery (Bradley & Mustard, 2006; Smith et al., 2022). While remote sensing using a time‐series of images has the potential to inform process‐based SDMs, it remains unclear whether this approach will improve forecasts of range shifts.

Because of the impacts of biological invasions (Fantle‐Lepczyk et al., 2022; Pyšek et al., 2020), there is a need to improve predictions of invasive species' potential distributions to inform management strategies (Hanley & Roberts, 2019; Pyšek et al., 2020). SDMs are an important tool for invasive species management, used to identify areas of previously undetected populations and predict suitable but currently unoccupied areas. Recently, remote sensing approaches have accurately detected populations of individual invasive plant species (Asner et al., 2008; Clinton et al., 2010; Dahal et al., 2022; Ren et al., 2021). Imagery time series are likely of particular value for detecting occurrences in remote, unsampled regions and for determining regions where new invasion is occurring (Müllerová et al., 2023). In a management context, leveraging remote sensing to predict occurrences beyond accessible areas at different points in time may help our ability to infer or forecast range dynamics (He et al., 2015; Wang et al., 2024).

In this study, we used a 21‐year dataset of Landsat imagery for Minnesota, USA, to quantify changes in the spatial distribution and population trends of leafy spurge (*Euphorbia virgata* Waldst. & Kit.; Euphorbiaceae; Levin & Gillespie, 2017), among the most economically damaging invasive plants in the United States (Fantle‐Lepczyk et al., 2022). To detect leafy spurge from imagery, we trained a land use/land cover (LULC) classification model using a temporal convolutional neural network (TempCNN), used a fully withheld testing dataset to evaluate classification accuracy, and interrogated the circumstances of classification failures. We then calculated population demographic trends over 21 years for each pixel and estimated the area of invasion across Minnesota through time. We also examined whether climatic variation during this period influenced detection and inferences about the extent of invasion. Last, to assess the potential utility of LULC species‐level predictions, we tested how SDMs built using remotely sensed predicted occurrence data compared to SDMs built using community science data. Further, we tested if incorporating remotely sensed inferences of population trends (i.e., process‐based modeling) improved model performance.

## MATERIALS AND METHODS

### Study system

Leafy spurge (*E. virgata* Waldst. & Kit.) is an herbaceous perennial plant that was introduced to North America from Eurasia in the 1800s. It has spread rapidly since introduction, now occupies nearly two million hectares of grasslands in the northern United States and southern Canada, and is among the most economically damaging invasive plants in the United States with losses between 1960 and 2020 totaling over $1 billion (Bangsund et al., 1999; DiTomaso, 2000; Dunn, 1979; Fantle‐Lepczyk et al., 2022). Plants emerge from dormancy in early spring before most herbaceous plants and flower in late spring (Lym, 1998). The flowers are subtended by bracts that have a distinct yellow‐green color. When damaged, plants produce a latex that can be toxic to livestock, which in turn affects the value of invaded rangelands (Messersmith et al., 1985).

Prior remote sensing studies have shown that the distinct spectral signal of its flowers is important for distinguishing leafy spurge from co‐occurring vegetation (Casady et al., 2005; Hunt et al., 2004; Lake et al., 2022; Mattilio et al., 2023; Stitt et al., 2006). Recently, the use of a temporal series of images has shown that the phenology of emergence, flowering, and senescence facilitates detection even with lower resolution imagery (Lake et al., 2022).

Leafy spurge was introduced to southwestern Minnesota in ca. 1890 and now occurs across a substantial fraction of Minnesota, USA (225,000 km^2^). By the 1930s, it had spread across the grasslands but occurrences in deciduous and boreal forests were uncommon (Hanson & Rudd, 1933). Leafy spurge remained rare in northern boreal forests until the 1990s.

### Developing and evaluating a remote sensing LULC classification algorithm

#### Landsat satellite imagery

We accessed Landsat 5 TM, 7 ETM+, and 8 OLI surface reflectance products (2000–2020) via Google Earth Engine (Gorelick et al., 2017). Landsat's 30‐m resolution and 16‐day revisit period are ideal for tracking vegetation dynamics (Vogelmann et al., 2016). We utilized spectral data from the visible (blue, green, red), near infrared, and shortwave infrared bands (SWIR 1 and 2), along with the Normalized Difference Vegetation Index [NDVI = (near infrared − red)/(near infrared + red)], a proxy for photosynthetic activity (Gamon et al., 1995; Young et al., 2017). We processed the imagery by removing pixels affected by cloud, cloud shadow, and radiometric saturation using quality assessment bitmasks. We then created three 2‐month seasonal windows per year (March–April, May–June, and July–August) corresponding to key events in leafy spurge's growing season: emergence, flowering, and senescence. Finally, we sampled the median pixel value across all available images for each spectral band within these seasonal windows (Figure 1; additional methods for image processing can be found in Appendix S1: Section S1.1).

**FIGURE 1:**
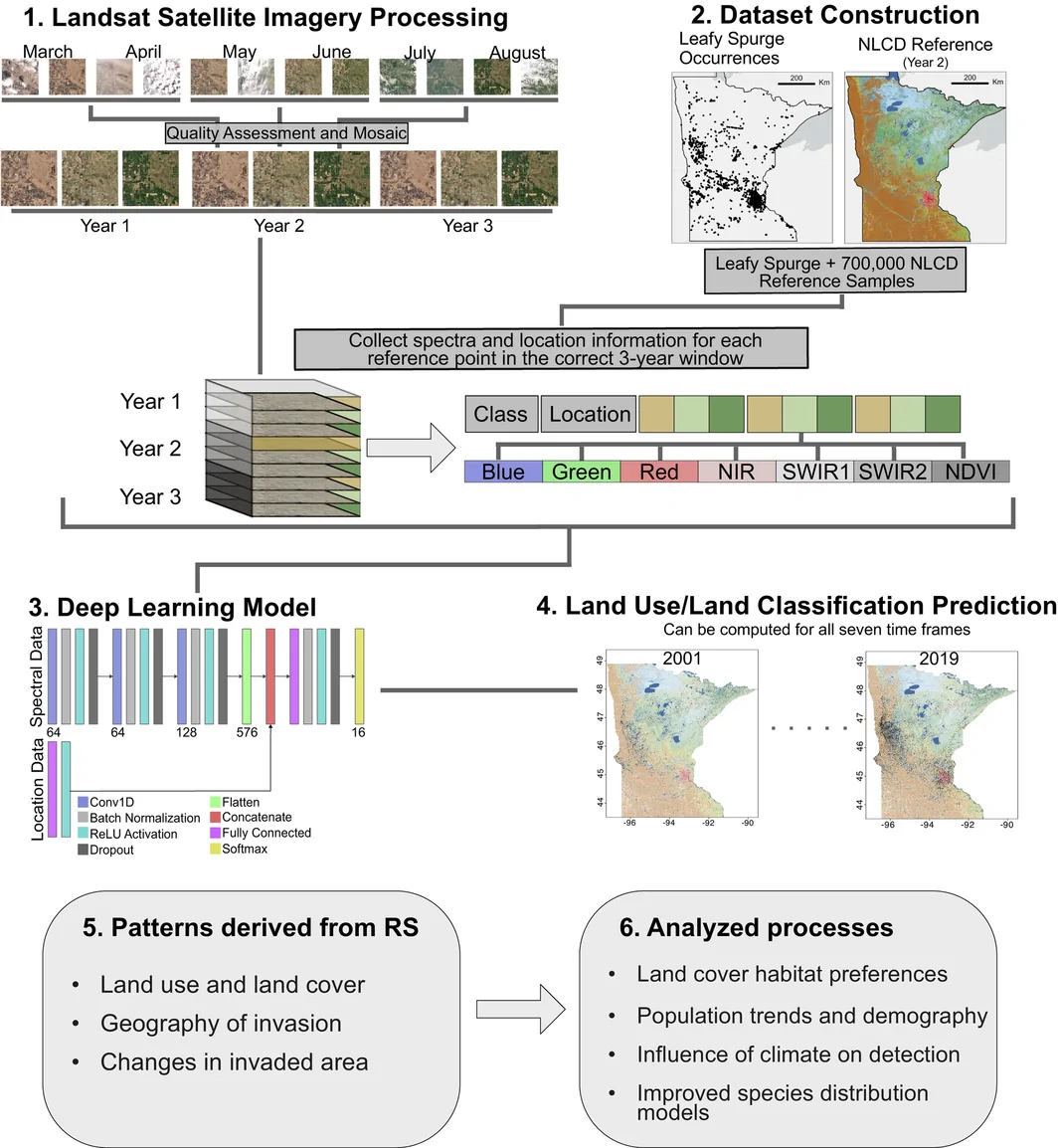
Overview of remote sensing models. (1) Landsat satellite image time‐series mosaics were processed with Google Earth Engine. Landsat imagery (2000–2020) included three 2‐month periods per year: March–April, May–June, and July–August. (2) We sampled all community science leafy spurge records available in public databases and 700,000 random points from the National Land Cover Database (NLCD) between 2000 and 2020 to construct a reference dataset used to train the land use/land classification model (LULC). The data vector for each reference sample included the land cover classification (leafy spurge or NLCD class), location (lat/lon), and spectral information from a 3‐year time frame centered on a temporal interval of interest. (3) A temporal convolutional neural network (TempCNN) architecture was used for LULC training to learn the relationship between land cover class and the 3 years of spectral data and location information. (4) After training and evaluating the TempCNN model, land cover classes were predicted across the state of Minnesota. (5) Patterns that can be derived directly from remotely sensed (RS) prediction maps. (6) Processes that can be analyzed using RS data.

#### LULC reference maps

We used the USGS National Land Cover Database (NLCD) classification mask to associate Landsat reflectance data with land cover classes (Yang et al., 2018). The NLCD is a vetted and ground‐truthed data product developed using Landsat imagery that provides pixel‐wise information on land cover classes (Yang et al., 2018). The NLCD for our study region included 15 land cover classes (e.g., open water, boreal forest; see: Appendix S1: Section S1.2) and was updated approximately every 3 years. We obtained historical NLCD maps from 7 years that fell within the 21‐year span of our study: 2001, 2004, 2008, 2011, 2013, 2016, and 2019.

#### Leafy spurge occurrence data

We downloaded occurrences between 2000 and 2020 from the Early Detection and Distribution Mapping System (EDDMapS.org) and iNaturalist (iNaturalist.org) for Minnesota (Appendix S1: Table S1). We filtered occurrences by removing duplicate, imprecise (less than 0.01 decimal degrees of precision), and implausible (e.g., over water) records. We kept only verified occurrences from EDDMapS and research‐grade occurrences from iNaturalist. We spatially thinned points to retain one occurrence per 100 m. In total, we obtained 3345 occurrences for use in downstream analyses (hereafter: community science occurrences). While representing a substantial effort, community science occurrences are unstructured and often include roadway and apparency bias.

We reclassified the existing NLCD maps based on the 3345 leafy spurge occurrences relative to their corresponding timeframe (e.g., if leafy spurge was reported in 2018, 2019, or 2020, we reclassified the pixel in the 2019 NLCD map; Appendix S1: Section S1.2; Table S1). These updated NLCD maps provided the reference pixels and spectral reflectance data used to train and validate the remote sensing LULC classification model.

#### TempCNN architecture and training data

We developed a LULC classification algorithm using a TempCNN architecture (Pelletier et al., 2019) and Landsat spectral data from 2000 to 2020 (Figure 1). A TempCNN is a supervised deep‐learning LULC algorithm that learns spectral and phenological patterns from time‐series imagery (Allred et al., 2021; Pelletier et al., 2019). For training and testing, we used spectral reflectance data drawn from Landsat imagery to develop a classification algorithm that could discriminate the phenological‐spectral profiles for the NLCD classes present in Minnesota plus leafy spurge.

To construct the core training and testing dataset, we divided the timeframe (2000–2020) into seven intervals of 3 years (2000–2002, 2003–2005, etc.) each corresponding to an NLCD classification map. For each time interval, we sampled all leafy spurge occurrences and 100,000 random locations from across the study area (Appendix S1: Tables S1 and S2). For each sampled pixel, we extracted land cover class (reclassified NLCD maps), latitude, longitude, and the Landsat spectral reflectance data from the specified time interval (*n* = 63; 7 spectral features/season × 3 seasons/year × 3 years; Figure 1). We repeated this process over the seven intervals from 2000 to 2020, yielding approximately 700,000 samples for model training (for additional details see Appendix S1: Section S1.3). This design ensured temporal overlap between field observations and satellite imagery and limited misclassification. However, some mislabeled samples were likely unavoidable due to rapid land cover change or under‐reporting of leafy spurge. Prior work suggests that low to moderate levels of label noise can be tolerated in deep learning and remote sensing classification (Pelletier et al., 2017; Rolnick et al., 2017).

The model architecture included a combination of 1D convolutional layers, dropout layers, and fully connected layers. Convolutional layers learned the spectral and phenological profiles of each land cover class, dropout layers minimized model overfitting, and fully connected layers passed information between layers. The normalized softmax layer produces a vector of pixel‐wise probabilities for each class (i.e., a single pixel summed across all softmax layers equals one). An argmax operation then assigns each pixel to the land cover class with the highest probability, resulting in the final predicted LULC map, which includes NLCD and leafy spurge classifications (for additional details see Appendix S1: Section S1.4).

#### TempCNN training, evaluation, and prediction

We split the dataset into training, validation, and testing sets using an 80/10/10 ratio (Appendix S1: Table S2). The model was trained for 100 epochs using the Adam optimizer and categorical cross‐entropy loss, with class weights applied to address data imbalance (Appendix S1: Section S1.5; Figure S1; Table S2). We then evaluated model performance on the testing set using a confusion matrix approach. For each class, we calculated overall accuracy, specificity (i.e., true negative rate), sensitivity (i.e., true positive rate), precision (i.e., the proportion of predicted positives that are correct), and the F2 score (accounts for model precision and sensitivity). We recognize that evaluation metrics are bound by the baseline accuracy of NLCD and leafy spurge labels, which is common for broad‐scale remotely sensed datasets. However, these metrics remain the most robust estimates of model performance (for additional details see Appendix S1: Section S1.6).

We applied the TempCNN to generate LULC predictions across Minnesota for the seven time intervals, analyzing approximately 250 million pixels per interval. For the leafy spurge class, we also extracted the normalized softmax layer at each interval. This process yielded a 30‐m resolution time series of leafy spurge landscape change and allowed us to identify high‐confidence leafy spurge predicted occurrences (hereafter “remotely sensed occurrences”; for additional details see Appendix S1: Section S1.7).

In addition to traditional confusion matrix‐based evaluations, we checked whether the means and distributions of softmax probabilities were similar for leafy spurge and NLCD classes. To do this, we used the model softmax outputs from our most recent prediction (2018–2020) for each land cover class and ground‐truthed it with an independent occurrence dataset for leafy spurge (2021–2023; *N* = 1018; Appendix S1: Table S1) not used for model training and the NLCD 2021 reference map (i.e., the 15 land cover classes, not including leafy spurge). For a subset of the 2021–2023 leafy spurge records (*N* = 940), population area (measured by the area of a spatial polygon and converted to square meters) was estimated by management professionals (Appendix S1: Figure S2). We characterized the distribution of leafy spurge softmax probabilities for recently reported occurrences to determine if new records were located in pixels with a high predicted leafy spurge probability. To quantify the extent to which small population size (or low densities) compromises the capacity to predict occurrences, we also examined whether the predicted probability of leafy spurge was associated with population area (*N* = 940) using quantile regression (for additional details see Appendix S1: Section S1.7). For other land cover classes, we randomly sampled 10,000 points per land cover class from the NLCD 2021 dataset and extracted the predicted probability values for these points from the associated softmax land class prediction. We visualized a histogram of probability values and estimated the median predicted probability for each land cover class (for additional details see Appendix S1: Section S1.7).

#### Identifying high‐confidence leafy spurge occurrences for total area invaded

To convert the continuous TempCNN softmax predictions into discrete predicted occurrences, we thresholded the leafy spurge normalized softmax layer at *p* = 0.5. The resulting pixels represent a collection of putative leafy spurge occurrences. This conservative threshold ensures that the predicted probability of leafy spurge at a pixel exceeds the combined probability of all other land cover classes. Although we acknowledge that these predictions may still retain some of the TempCNN labeling uncertainty, this criterion is stricter than both the standard argmax classification and the *p* = 0.38 value needed to achieve 75% leafy spurge sensitivity relative to community science observations (Appendix S1: Figure S3). This thresholded dataset was then used to determine the geography and timing of leafy spurge occurrences for subsequent analyses of roadside association.

#### Extent of invasion and climate trends

We estimated population change from 2000 to 2020 for individual pixels using linear regression of normalized softmax probability of leafy spurge for seven intervals from 2000 to 2020 on year. The slope of the regression for each pixel served as a proxy for the rate of population change. Positive slopes (>+0.5 SD from mean) were taken to indicate growth, negative slopes (< −0.5 SD from mean) were taken to indicate decline, and slopes near zero indicated stability or fluctuations through time.

We tested the hypothesis that climate variation causes the TempCNN model to predict fewer invaded pixels due to reduced plant biomass and flower production. We used the Palmer Drought Severity Index (PDSI) (Palmer, 1965), which captures variation in temperature, precipitation, and soil moisture. We averaged PDSI values over the 3‐year intervals used for the TempCNN and partitioned the data into nine geographic regions corresponding to NOAA's climate divisions. We then quantified invaded area for each region and timeframe as described above. We tested whether predicted invasion extent (number of positive leafy spurge pixels) was related to drought severity and geographic region using a general linear model. Due to data overdispersion, we specified the model family as “quasipoisson” using the glm function. We assessed the significance of model terms using analysis of deviance with χ^2^ tests (“Anova” function, “car” package) and quasi‐*F* tests (“anova” base R function) (Dunn & Smyth, 2018).

### Geographic patterns and timing of invasion

#### Geography and timing of leafy spurge occurrences, expansion, and extirpation

We examined the geography and timing of remotely sensed occurrences, extirpations, and continually present populations from 2000 to 2020. We categorized new occurrences into two time periods: early occurrences and late occurrences. We defined early occurrences as pixels where leafy spurge was absent in the first two prediction intervals (i.e., 2000–2002 and 2003–2005) but present in all subsequent five periods (i.e., 2006–2006 through 2018–2020). Similarly, we defined late occurrences where leafy spurge was absent in the first five prediction intervals and present in the last two periods. Extirpations included pixels where leafy spurge was present in the first two predictions and absent in all five subsequent periods. Continually present populations were those pixels where leafy spurge remained present across all seven predictions from 2000 to 2020. For all categorizations, we used the 0.5 threshold for positive presences and a conservative threshold for absence (<0.15).

#### Testing for roadside association

Roadside surveys are a major contributor to sampling bias in occurrence datasets (Geurts et al., 2023). To estimate the potential for roadside association in our datasets, we measured the distance to primary and secondary roads for both community science occurrences and remotely sensed occurrences (*N* = 1771, Appendix S1: Sections S1.8 and S1.9 for details) using the U.S. Census Bureau's MAF/TIGER Database (Appendix S1: Section S1.8 for details). We used a k‐d tree nearest neighbor search to calculate the shortest distance between occurrence points and road segments. For comparison, we generated a null expectation by calculating distances to the nearest road for an equal number of randomly selected points (*N* = 999 permutations) within Minnesota. We compared the mean distance to roads for both occurrence sets and determined if either value was less than random expectation at the 0.05 level of significance (one‐tailed test).

### Species distribution modeling

We constructed climate SDMs to compare the performance of models built using community science occurrences and remotely sensed occurrences. We further supplemented our remotely sensed occurrence‐based models with process‐based information derived from the TempCNN (weighting by temporal changes in softmax probability). To match the number of community science occurrences (*N* = 1771), we generated a set of 10 subsampled remotely sensed occurrence datasets (see Appendix S1: Section S1.10 for details). For remotely sensed datasets, we also generated two weighting values for each occurrence based on the TempCNN softmax probability (e.g., high softmax probability caused by high density), and the rate of change in population density (slope; e.g., increasing softmax probability caused by increasing density or new occurrence). Using these data, we evaluated four SDM model types based on differing occurrence inputs: (1) unweighted community science presence, (2) unweighted remotely sensed occurrences, (3) remotely sensed probability‐weighted occurrences, and (4) remotely sensed slope‐weighted occurrences. For all models, we used three standard bioclimatic variables (WorldClim: Bio 10: mean temperature of the warmest quarter, Bio 11: mean temperature of the coldest quarter, and Bio 12: annual precipitation; Appendix S1: Figure S4). Because we were interested in projections of habitat suitability, we built SDMs using two commonly used approaches, Maxent and Boosted Regression Trees with a range of hyperparameter combinations (Elith et al., 2008; Kass et al., 2021; Phillips et al., 2017). We projected models across Minnesota to compare the geography of habitat suitability and visualized the variable response curves to assess how the use of remotely sensed occurrences changed the predicted climate niche.

Overall, model performance was evaluated using fourfold cross‐validation to assess discrimination area under the curve (AUC) and regularization (AUC difference, or overfitting). Because these metrics rely, in part, on predicted labels from the TempCNN, we also calculated sensitivity (true positive rate) using the 2021–2023 validation occurrence dataset. These data were collected independently of the training data (Appendix S1: Figure S7). We calculated sensitivity for the entire test region and nonurban areas separately to evaluate omission errors. We used linear mixed models to test for differences in evaluation metrics where model type was treated as a fixed effect and dataset identity (e.g., subsample id) was treated as a random effect (see Appendix S1: Section S1.10 for more details).

## RESULTS

### Remote sensing accurately classified leafy spurge and land cover classes

The TempCNN predicted leafy spurge (observed) occurrences with high overall accuracy (97%) and high specificity (97%), comparable to other classes (Appendix S1: Table S3). However, the class showed moderate sensitivity (65%) and low precision (11%), resulting in an F2 score of 32%. The low precision stemmed from false positives generally reclassifying grassland/herbaceous, pasture/hay, and low‐intensity developed land cover classes (Appendix S1: Figure S8). Crucially, these are the habitats where leafy spurge is expected, suggesting a portion of these false positives are likely unreported leafy spurge populations.

We found agreement between our predicted land cover classes from 2018 to 2020 and the 2021 NLCD dataset, indicating that the TempCNN generalized beyond the training data. Softmax predicted probabilities for all classes were relatively high for NLCD 2021 and leafy spurge validation datasets (Appendix S1: Figure S9). Recent leafy spurge occurrences (2021–2023) were also well captured by the TempCNN. From the softmax probability layer predictions, the median probability of leafy spurge occurrences was 48%, which was similar to the median predicted probabilities of other classes in the TempCNN model—for example, deciduous forests (46%) and developed high‐intensity (54%) land cover classes (Appendix S1: Figure S9).

TempCNN predicted probability was high across the independent validation dataset (all means >0.80; all medians >0.92; Figure 2). However, quantile regression revealed that small populations had significantly greater variance in detection probability than larger populations. This variance was demonstrated by the median quantile showing a high intercept (0.92) and shallow slope (0.002), while the 5% and 25% quantiles exhibited much lower intercepts (0.19 and 0.73) and steeper slopes (0.23 and 0.006, respectively; Figure 2).

**FIGURE 2:**
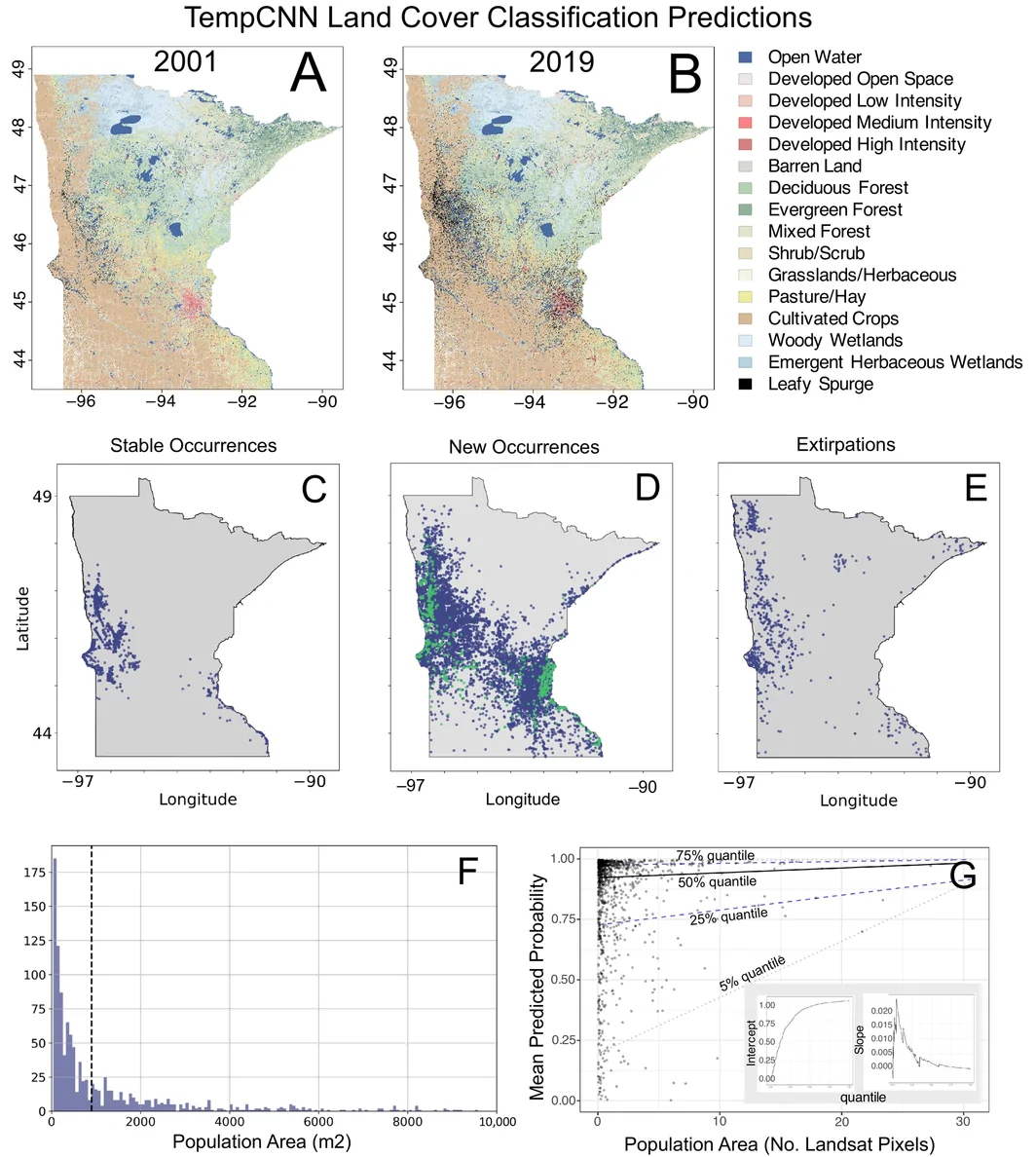
(A, B) Land classification prediction for (A) 2001 and (B) 2019 time period predicted by the temporal convolutional neural network (TempCNN). Land classifications are indicated by different colors with pixels predicted to be leafy spurge shown in black. (C–E) TempCNN predictions of temporal change in leafy spurge distribution. (C) Stable predicted occurrences from 2000 to 2020; predicted with ≥0.5 probability at all time periods (*N* = 3358 points). (D) New predicted occurrences. Green points appeared by 2006–2008 (*N* = 1286 points). Blue points appeared after 2014 (*N* = 17,609 points). (E) Extirpated populations were present in 2000–2005 but absent after (*N* = 4918 points). (F) Population area distribution of independent leafy spurge validation occurrences collected from 2021 to 2023. (G) Relationship between predicted probability of leafy spurge from the TempCNN model and population area, presented as number of pixels (one pixel = 900 m^2^). The lines represent quantile regression relationships. The inset shows the relationship between the quantile modeled using quantile regression and the intercept (left) and slope (right) of those relationships.

### Temporal dynamics of invasion and the effects of drought on remote sensing predictions

The invaded area expanded significantly from 1067 km^2^ in 2000–2002 to 7156 km^2^ in 2018–2020 (Appendix S1: Table S4). However, the extent also fluctuated, notably declining in 2009–2011 (3124 km^2^) and 2012–2014 (1372 km^2^) before increasing again. Invasion extent was significantly influenced by both drought severity (10.2% of model deviance) and region (84.7% of model deviance; Appendix S1: Table S5). Greater drought severity was associated with reduced detection by the TempCNN (and lower invasion extent; Figure 3), with the strongest relationship observed in west‐central and northwest Minnesota. These findings suggest that climate variation influences leafy spurge growth and flowering, which directly affects our capacity to detect it from satellite images.

**FIGURE 3:**
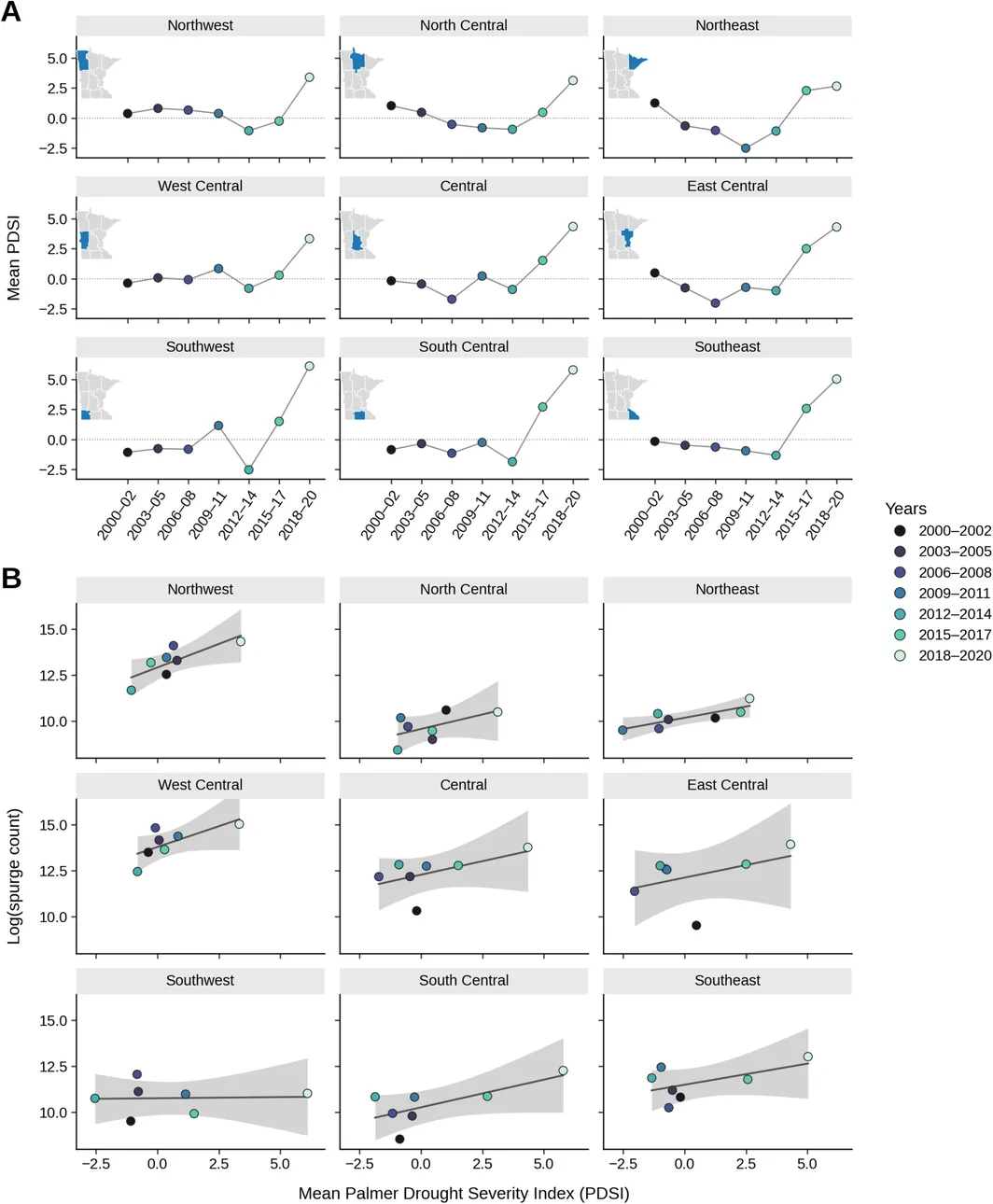
Regional climate variation shapes the predicted extent of leafy spurge invasion across Minnesota. (A) Mean Palmer Drought Severity Index (PDSI) values averaged over 3‐year periods from 2000 to 2020 for each climate division. Negative values indicate drought conditions, while positive values reflect wetter periods. The upper left corner of each graph provides a map that indicates the location for each of the nine climate divisions in Minnesota, which are defined by the National Oceanic and Atmospheric Administration's National Centers for Environmental Information. (B) Relationship between leafy spurge invasion extent and mean PDSI, with data faceted by climate division. The *y*‐axis shows the log‐transformed invasion extent predicted by the temporal convolutional neural network (TempCNN) model; the *x*‐axis shows mean PDSI values across corresponding 3‐year periods.

### Geography and timing of new occurrences and extirpations

Leafy spurge was already well established in Minnesota prior to the timeframe of our study. Populations were most abundant in the prairies and grasslands of west‐central Minnesota, with a smaller but notable presence in urban areas (Figure 2). In the timeframe of our study, we observed new areas of invasion across most of the state except the far northern and southern regions (Figure 2). Between 2000 and 2010, newly predicted populations were primarily concentrated in prairies/grasslands near urban areas on the western (Fargo‐Moorhead) and eastern sides of the state (Minneapolis‐St. Paul). Between 2010 and 2020, substantially more spatial spread radiated from urban to rural areas, including many new occurrences in central Minnesota. Extirpations were primarily detected in western areas but were absent from urban areas (Figure 2).

### Population trends of leafy spurge through time

For individual pixels, the mean slope of the relationship between leafy spurge probability and time was 0.003 (std dev: 0.001), indicating an overall increase in leafy spurge across Minnesota (Figure 4; Appendix S1: Table S4). We observed population growth in 13.1% of the state (>+0.5 SD from mean). Population growth was located primarily in urban areas (i.e., Minneapolis‐St. Paul and Fargo‐Moorhead) and prairie or grasslands (i.e., west‐central Minnesota) (Figure 3). We observed population decline in 8.4% of the state (<−0.5 SD from mean). Population decline was located primarily in the boreal region (i.e., northeastern Minnesota) and portions of western Minnesota (Figure 4).

**FIGURE 4:**
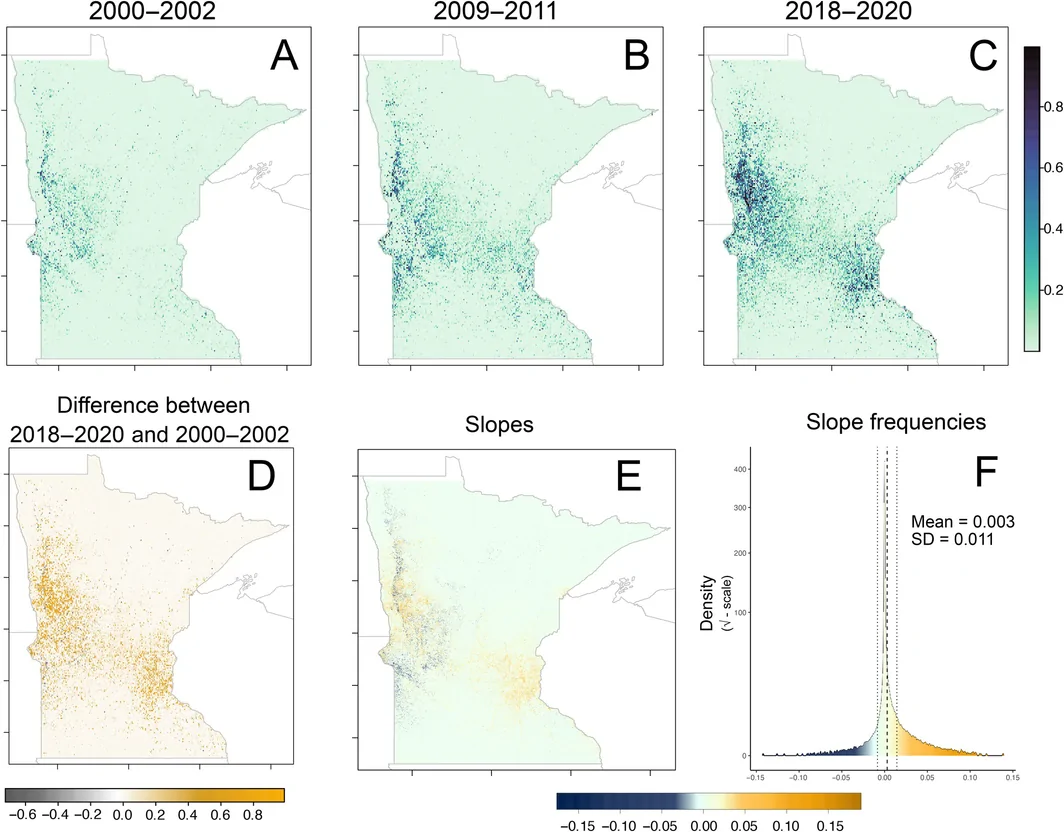
Predicted probability and population dynamics of leafy spurge for individual pixels from the temporal convolutional neural network (TempCNN). (A–C). Probability of leafy spurge for (A) 2000–2002, (B) 2009–2011, and (C) 2018–2020. (D) The difference between the 2018–2020 and 2000–2002 prediction. Orange pixels indicate positive values and gray pixels indicate negative values. (E) Slope values from regressions of predicted probability versus time period. (F) Histogram of the slope values. The dashed line indicates the mean slope and the dotted lines are ±SD.

### Remotely sensed occurrences have less associated with roadsides

Remotely sensed occurrences and community science occurrences exhibited only moderate spatial overlap (Appendix S1: Section S2.1). Remotely sensed occurrences were more likely to be found near roads than random expectation (Figure 5; *p* < 0.001). Remotely sensed occurrences were an average of 3.7 km from roads and 50% of occurrences were within 3 km of a road (Figure 5). Community science occurrences were even more likely to be associated with roads than remotely sensed occurrences (Figure 5). On average, community records were 2.6 km from roads and 50% of occurrences were within 1 km of a road. This result indicates spatial bias in the community science dataset that is likely due to sampling.

**FIGURE 5:**
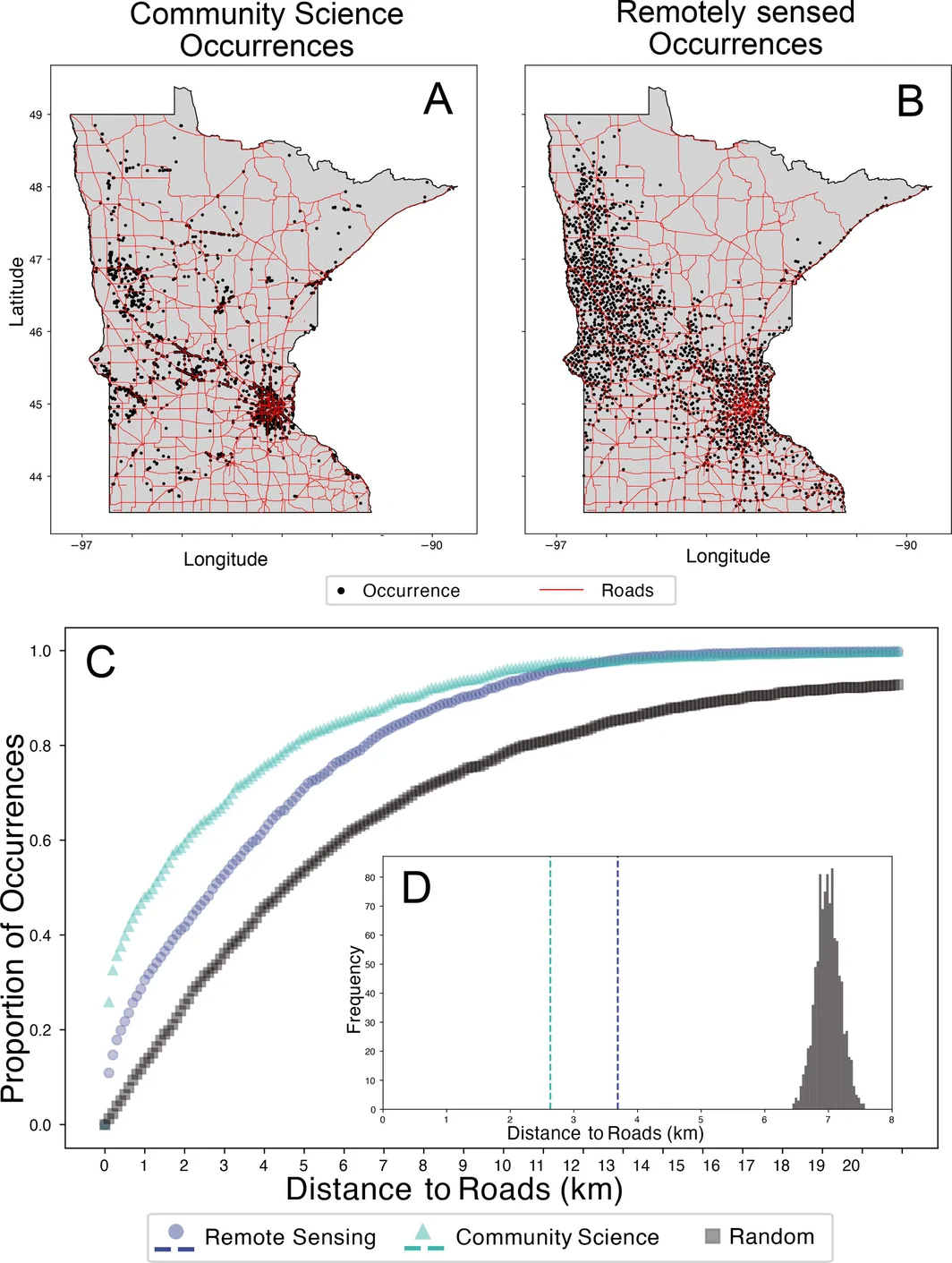
Leafy spurge occurrences used for testing spatial sampling bias and species distribution model (SDM) development. (A, B) Mapped occurrences (black dots) and major roads (interstates and highways; red lines). (A) Community science occurrences from 2000 to 2020 (*N* = 1771). (B) Random sample of remotely sensed predicted occurrences (*N* = 1771) from the temporal convolutional neural network (TempCNN). (C) Relationship between the cumulative proportion of occurrence records and distance to the nearest road in kilometers. (D‐inset) Comparison of the mean distance to a roadway for community science occurrences (2.63 km), remotely sensed predicted occurrences (3.69 km), and a distribution of randomly drawn occurrences (*N* = 999).

### Remotely sensed occurrences improved SDM accuracy especially in nonurban regions

Models fit using remotely sensed occurrences performed better overall than models fit using community science occurrences (Figure 6). We report the Maxent results only for simplicity, but BRT results were similar and are reported in Appendix S1: Section S2.2; Table S6. Remotely sensed models had higher discrimination scores (reported values are mean ± SE; AUC: community science: 0.783 ± 0.003; remotely sensed: unweighted: 0.797 ± 0.001, probability‐weighted: 0.799 ± 0.001, slope‐weighted: 0.812 ± 0.001) and were less likely to be overfit (AUC difference; community science: 0.054 ± 0.002; remotely sensed: unweighted: 0.015 ± 0.001, probability‐weighted: 0.006 ± 0.001, slope‐weighted: 0.006 ± 0.001). In addition, remotely sensed models had consistently higher sensitivity in chronically undersampled nonurban areas (community science: 0.497 ± 0.01; remotely sensed: unweighted: 0.648 ± 0.003, probability‐weighted: 0.617 ± 0.003, slope‐weighted: 0.606 ± 0.003). Weighting generally only affected the relative order and variance among model metrics and did not change the overall performance advantage gained from using remotely sensed occurrences (Appendix S1: Figure S10).

**FIGURE 6:**
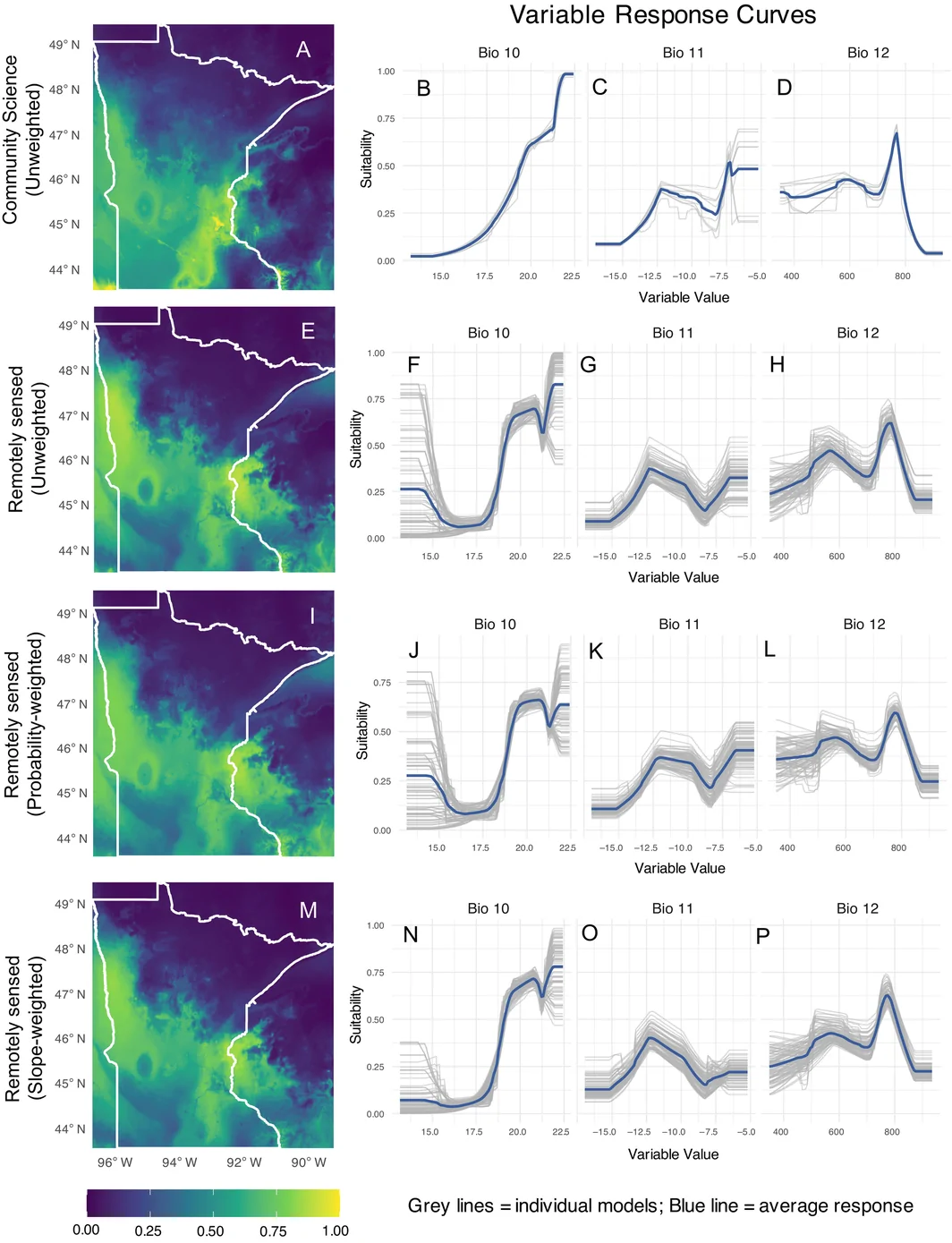
Maxent model projections of climate suitability and variable response curves. Left column panels are mean model climate suitability projections from models using: (A) unweighted community science occurrences, (E) unweighted remotely sensed occurrences, (I) probability‐weighted remotely sensed occurrences, (M) slope‐weighted remotely sensed occurrences. Each model projection is the average of models built with different combinations of hyperparameters and subsample datasets (*n* = 9 for community science, *n* = 90 for remotely sensed). Right column panels are variable response curves for the three environmental variables (Bio 10: mean temperature of the warmest quarter, Bio 11: mean temperature of the coldest quarter, Bio 12: annual precipitation) from Maxent models. Gray lines are individual model curves and blue lines are the average across all model curves. (B–D) Variable response curves for models using unweighted community science occurrences, (F–H) unweighted remotely sensed occurrences, (J–L) probability‐weighted remotely sensed occurrences, (N–P) slope‐weighted remotely sensed occurrences.

While model response curves shared similar shapes across central environmental ranges, curves showed marked differences at the edges of the niche space, indicating that remotely sensed occurrences may occur in more extreme and less well‐sampled parts of the state (Figure 6). These relative differences were most pronounced at lower values of temperatures during the warmest quarter (Bio 10) and at higher values of annual precipitation (Bio 12). In projections, this translated to predictions of relatively higher suitability in the northwest regions of Minnesota (Figure 6).

## DISCUSSION

In this study, we used a 21‐year time series of Landsat imagery coupled with deep learning to detect leafy spurge and assess spatial and population changes over 225,000 km^2^ in Minnesota, USA. Our analyses show that the area invaded by leafy spurge increased by as much as 570% over 21 years. Invasion during this period involved range expansion and range infilling in two distinct areas in Minnesota that differ in climate. Remotely sensed occurrences were closer to roadsides on average, albeit less strongly associated than community science occurrences. These results suggest that factors other than climate (e.g., disturbance) may be particularly important in driving invasion. Including remotely sensed predicted occurrences and demographic estimates of population growth in SDMs reduced overfitting and improved predictions compared to models using community data alone. The greatest improvement in model fit occurred in the western portion of the study region and near urban centers, where growth rates were highest. Our findings indicate that remote sensing can monitor invasion, identify spatial bias in observed and predicted occurrences, and improve SDMs.

### Factors modulating detection and inferences about invasion

Leafy spurge was detectable from satellite imagery due to its distinct phenology (emergence, flowering, and senescence) and spectral signal (yellow‐green flowering bracts) (Hunt et al., 2004). While previous remote sensing surveys examined 10 to <1000 km^2^ (Hunt & Parker Williams, 2006; Lake et al., 2022; Mattilio et al., 2023; Mitchell & Glenn, 2009; Mladinich et al., 2006; Stitt et al., 2006) or leveraged submeter resolution or hyperspectral data (Hunt et al., 2010; Lake et al., 2022; Parker Williams & Hunt, 2002), our study leveraged the deep temporal catalogue of (lower resolution) Landsat satellite imagery to detect leafy spurge over broad spatial scales with high agreement to labeled testing data (97%). Evaluating the model using ground truth data from publicly available datasets indicated that our TempCNN was effective across hundreds of thousands of square kilometers. Over the two decades of our study, models predicted a substantial increase in the area invaded (ca. 100,670–715,600 ha). These estimates are likely conservative because we used a relatively strict threshold for predicted occurrences (*p*_leafy spurge_ = 0.5) that prioritized high confidence in classification assignment from our LULC/TempCNN model. We chose that threshold to reduce false positives but acknowledge that this choice causes populations with a lower probability to be excluded from estimates of invaded area (i.e., causes false negatives). However, we do not expect that leafy spurge classification errors affected the spatial and temporal trends predicted by our model. When we used a different probability threshold for leafy spurge (e.g., *p* = 0.38, the 75% sensitivity threshold for the validation dataset), spatial and temporal patterns of invasion were very similar to those reported for *p* = 0.5. The patterns that we observed also align with observations by researchers and managers in this region and changes since previous estimates were reported (e.g., Skinner et al., 2002).

Although our TempCNN had high accuracy in detecting leafy spurge, the model still predicted false positives and false negatives as evidenced by moderate F2, precision, and sensitivity scores. False positives often occurred in areas with high‐quality habitat not currently identified as occupied (e.g., grasslands adjacent to existing leafy spurge populations). False positives could also arise if our model confused leafy spurge with other plants that have a similar spectral profile, or if the initial land classification map was incorrect (Stehman & Foody, 2019). Because our prior work has shown that phenology improves specificity for leafy spurge (Lake et al., 2022), it is less likely that false positives are due to misidentification of plant species. Conversely, false negatives may occur because our model was less likely to predict small and low‐density populations (Figure 2). Future studies could reduce false negatives by incorporating higher spatial resolution imagery such that smaller patches of leafy spurge were more easily distinguishable from background vegetation. For example, combining Landsat and Sentinel‐2 products (i.e., Harmonized Landsat/Sentinel‐2) would increase the resolution and frequency of reimaging (Pastick et al., 2020; Zhou et al., 2019). However, Sentinel‐2 images are available only after 2015 and therefore were not feasible for estimating spatial and long‐term demographic trends in this study.

Despite an overall trend of increasing area invaded by leafy spurge through time, we observed substantial fluctuations. Those fluctuations were predicted by the severity of drought: Overall area invaded was lower in years of drought and higher in years where precipitation was average or above average. Given that accurate detection depends on both the spectral signal of inflorescences and phenology, it is likely that drought limited detection because flowering was less likely and biomass was reduced in drought years. For example, northwest Minnesota experienced severe drought in 2012–2014 and the predicted area invaded by leafy spurge was reduced by 83% compared to the years immediately prior (2009–2011). After the drought ended (2015–2017), the predicted leafy spurge area rebounded by 447% (Figure 4).

Importantly, single timeframe declines in predicted area do not necessarily imply true extirpation. Leafy spurge populations can persist below detectable levels via the existence of extensive perennial underground rhizomes from which aboveground shoots can regenerate quickly when environmental conditions become favorable again (Selleck et al., 1962). While this biological response to drought affected detection, it also revealed that remote sensing could assess plant performance in relation to environmental variation. Likewise, multiple years of evidence are likely important for verifying eradication. Overall, time‐series data help to buffer against making faulty inferences during unusual years, but environmental variation must still be accounted for given how strongly it can influence detection.

### Roadside association and management implications

Leafy spurge occurrences were more likely to occur near roads than expected at random for both the TempCNN‐based predicted occurrences and the community science dataset (Figure 5). The stronger roadside clustering in the community science records is likely due to accessibility and observer bias (Kadmon et al., 2004); this type of spatial association can influence the occurrence‐environment relationships inferred by SDMs (Inman et al., 2021). However, the persistence of a roadside signal in the remotely sensed occurrences suggests that the pattern is not solely an artifact of observer bias. Roadsides are often more open and subject to high disturbance, which are habitat characteristics favored by leafy spurge. In addition, roadsides may function as dispersal corridors for invasion. Roadside associations have been reported for many invasive plants (Parendes & Jones, 2000; Tyser & Worley, 1992), including leafy spurge. For example, Huerd and Taylor (1998) reported that many leafy spurge populations in Iowa occurred along roadsides and suggested that invasive spread may be facilitated by contaminated soils used in road construction, transport of contaminated hay, roadside mowing, and subsequent dispersal of seeds into adjacent fields. Our results are consistent with roadways as a pathway of invasion for leafy spurge. For management, our results suggest that roadsides are important targets for monitoring and eradication.

### Remote sensing of population trends

Understanding demographic variation across species' ranges is important for determining the causes of range limits and factors modulating range shifts in response to climate change (Angert et al., 2011; Eckhart et al., 2011; Villellas et al., 2013). However, such datasets require enormous effort over long time periods and are not feasible for most systems. Our study suggests that remote sensing is a viable alternative in some systems for quantifying population trends and documenting range expansion and infilling. In this study, we used linear regression (e.g., slope coefficient) to characterize if populations were increasing, decreasing or lacked directional change through time. Positive slopes were concentrated in west‐central grasslands and in the Twin Cities region; both regions also had the highest concentrations of recent occurrences in community science datasets (e.g., our 2021–2023 validation dataset). These results suggest continued expansion and infilling in two different contexts: grassland regions of west‐central Minnesota where leafy spurge is already well established and urban or roadside landscapes where disturbance may facilitate spread. By contrast, negative slopes were concentrated in the western portion of the study region, where biological control of leafy spurge has been widely implemented (Skinner et al., 2006). The biogeographical pattern of declines in regions with longstanding biocontrol suggests that *Aphthona* flea beetles are effective at population suppression in some cases. Thus, our inferences of population trends is consistent with independent ecological observations of both ongoing spread and local decline. Although our data cannot fully isolate biocontrol effects from other management actions or land use change, merging remote sensing with records of biocontrol attempts is a fruitful avenue for future research.

Our estimates of population change may have also been affected by factors unrelated to habitat suitability. Where leafy spurge occurs in small or low‐density populations, we may have failed to predict it in some years, especially during drought. Although the variance in predicted probability for small populations was relatively high, the median predicted probability was also high, suggesting that many small populations were still detected. In addition, conversion of grasslands to crops and invasive species management (e.g., herbicides, prescribed fire, and biocontrol applications) may have caused local declines unrelated to climatic suitability alone. These influences likely contributed to some local trends but are unlikely to explain the broad regional patterns we observed.

### SDMs predict greater suitability in rural and extreme environments

SDMs built using remotely sensed data were more regularized with better discrimination than those built using the community science dataset. Regardless of the model algorithm used, the remotely sensed models had higher model discrimination (AUC) and were less overfit (AUC difference) than the community science models. This was reflected in changes to variable response curves, which indicated higher suitability in areas of low summer temperatures and higher precipitation. For example, remotely sensed SDMs predicted much higher habitat suitability in southeastern and western Minnesota compared to the community science models (Figure 6; Appendix S1: Figure S10). The community science models predicted highest suitability in primarily urban or disturbed (i.e., Interstate 35 in southern Minnesota) areas, where observation bias is known to be strong. Because invasive species are often at nonequilibrium with respect to geography, SDM predictions may be most reliable over short time periods (Hui, 2023). As invasions approach equilibrium, SDMs may more accurately predict distributions over longer time periods (Briscoe Runquist et al., 2019; Guareschi et al., 2024). For species with rapidly changing distributions, SDMs should be reassessed over time using updated predicted occurrence datasets.

Using information on population trends to build models did not substantially change SDM performance or the geography of habitat suitability. This result is not surprising given that the geography of occurrences is strongly correlated with the geography of positive slopes. In future studies with finer grained temporal resolution, it may be possible to incorporate additional complexity (i.e., density‐dependence) to refine inference of population trends. Such demographic insights may be particularly useful for improving SDMs but may require caution as demography and occurrence probability may not be positively correlated (Thuiller et al., 2014).

## CONCLUSIONS

Our study expands remote sensing of an individual species to a large geographic scale and over a long time series despite modest resolution of imagery. Our maps show that the increase in new records has been particularly rapid over the past 10 years and that the spread has been mediated by roadways. We also observed rapid population expansion (and some range expansion) that was concentrated in two disjunct regions near metropolitan areas that differ in climate. Remotely sensed occurrences were less spatially biased than community science occurrences and produced more accurate SDMs. However, the persistence of roadside association in remotely sensed occurrences suggest that disturbance may be important to invasion and that roadways act as invasion pathways. More comparisons of this sort are needed to evaluate the extent to which publicly available occurrence datasets differ from remotely sensed predictions and whether these differences have consequences for SDMs. Overall, examining the population trends of individual species over large geographic areas and long time series is feasible and should become more tractable as higher resolution and high‐cadence satellite imagery (e.g., Sentinel, Planetscope) imagery accumulates.

## AUTHOR CONTRIBUTIONS

Thomas A. Lake, Ryan D. Briscoe Runquist, and David A. Moeller conceived of and designed the research study. Thomas A. Lake collected data for and developed remote sensing models. Ryan D. Briscoe Runquist collected data for and developed species distribution models. Thomas A. Lake and Ryan D. Briscoe Runquist performed the analyses. Thomas A. Lake, Ryan D. Briscoe Runquist, and David A. Moeller contributed substantially to the drafting, editing, and final version of the paper.

## CONFLICT OF INTEREST STATEMENT

The authors declare no conflicts of interest.

## Supporting information


Appendix S1.

## Data Availability

Data (Lake et al., 2026) are available through the Data Repository for the University of Minnesota at https://doi.org/10.13020/ba6e-yt29. Code (Lake, 2026) is available in Zenodo at https://doi.org/10.5281/zenodo.20315228.
