# Supplementary material for: Two decades of Landsat images reveal spatial and temporal dynamics of invasion and improve species distribution models
Source: Ecol Appl. 2026 Nov 4;36(7):e70331. doi: 10.1002/eap.70331 (PMC13735486; doi:10.1002/eap.70331)
Supplement: Supplementary file 1 [file EAP-36-e70331-s001.pdf]

Appendix S1

Two decades of Landsat images reveal spatial and temporal dynamics of invasion and improve species distribution models

Thomas A. Lake, Ryan D. Briscoe Runquist, and David A. Moeller

Ecological Applications

Table of Contents

Section S1: Methods 4

Section S1.1 Landsat satellite imagery 4

Section S1.2 Ground truth maps 4

Section S1.3 TempCNN training data 4

Section S1.4 Temporal convolutional neural network (TempCNN) architecture 5

Section S1.5. TempCNN model training 5

Section S1.6 TempCNN model evaluation 6

Section S1.7 TempCNN model predictions 7

Section S1.8 Spatial overlap and autocorrelation of occurrence datasets 8

Section S1.9 Assessing spatial bias near roadsides 8

Section S1.10 Species Distribution Models 8

Section S1.10.1 Environmental Data 8

Section S1.10.2 Occurrence sampling for SDMs 9

Section S1.10.3 Occurrence weighting for process-based models 9

Section S1.10.4 Modeling algorithms 9

Section S1.10.5 Model evaluation metrics 10

Section S2: Results 11

Section S2.1 Spatial biases in occurrences 11

Section S2.2 Boosted Regression Tree (BRT) results 11

Section S3: Supplementary Figures 12

Figure S1. Training history of the temporal convolutional neural network (TempCNN). 12

Figure S2. Occurrence points and polygon records of leafy spurge in Minnesota. 13

Figure S3. Sensitivities of CS occurrences. 14

Figure S4. Bioclimatic variables used for species distribution modelling. 15

Figure S5. Cross-validation spatial blocks for species distribution models using community science and remote sensing occurrences datasets. 16

Figure S6. Binary map of the predicted probability of leafy spurge. 17

Figure S7. Independent records of leafy spurge from 2021-2023 partitioned by geographic region. 18

Figure S8. Land Classification Masks with Confusion Matrix of True Positive Rates 19

Figure S9. Comparison of predicted land cover class probabilities from our latest prediction (2018-2020) with the National Land Cover Database's 2021 dataset. 20

Figure S10. Comparison of species distribution model evaluation metrics. 21

Figure S11. Boosted regression tree model predictions of climate suitability and partial response curves 22

Section S4: Supplementary Tables 23

Table S1. Community science occurrence data of leafy spurge from 2000 to 2023. 23
Table S2. Number of training, validation, and testing records. 24
Table S3. Temporal convolutional neural network model performance and evaluation metrics. 25
Table S4. Area of leafy spurge invasion in square kilometers predicted from the TempCNN model between 2000 and 2020. 25
Table S5. Sequential analysis of deviance table for Palmer Drought Severity Index. 26
Table S6. ANOVA tables to test for difference in four SDM evaluation metrics. 26
References 27

Section S1: Methods

Section S1.1 Landsat satellite imagery

We obtained Landsat 5 TM, 7 ETM+, and 8 OLI surface reflectance products (Collection 2 Tier 1) from 2000 to 2020 via the Google Earth Engine platform. We used spectral data from the visible (blue, green, red), near infrared, and shortwave infrared bands (SWIR 1 & 2) in addition to the normalized difference vegetation index [NDVI = (near infrared - red) / (near infrared + red)]. We began by removing pixels with cloud, cloud shadow, and radiometric saturation using quality assessment bitmasks ('QA_Pixel' and 'QA_Radsat'). After cleaning the data, we created image mosaics for two-month periods by calculating the median pixel value for each spectral band. This process yielded a Landsat time-series dataset composed of seasonal image mosaics from 2000 to 2020, one from each of the following two-month periods: March-April, May-June, and July-August.

For instance, if six Landsat images captured between March and April in one year have pixel values for the 'blue' band as (0.51, 0.55, 0.62, NA, 0.62, 0.73), the NA values indicate data removed during quality assessment. We then calculated the median value for each spectral band and pixel to construct the seasonal mosaic using the 'ee.ImageCollection.median' function in Google Earth Engine. We repeated this process to create mosaics for March-April, May-June, and July-August across all available images for each year from 2000 to 2020.

Section S1.2 Ground truth maps

We used the National Land Cover Database (NLCD) to associate Landsat pixels with land cover classes for classification. The NLCD provides a 30-meter resolution classification of 20 land cover classes across the conterminous US for nine years (2001, 2004, 2006, 2008, 2011, 2013, 2016, 2019, and 2021). We used pixel-based reclassification to include leafy spurge occurrence points (N = 3,345) into the existing NLCD maps based on reported survey dates (Table S1). For example, leafy spurge occurrences reported in 2018, 2019, and 2020 were added to the nearest NLCD map (2019) by reclassifying existing land cover types.

The 15 NLCD land cover classes present in Minnesota include: Open Water, Developed Open Space, Developed Low Intensity, Developed Medium Intensity, Developed High Intensity, Barren Land, Deciduous Forest, Evergreen Forest, Mixed Forest, Shrub/Scrub, Grasslands/Herbaceous, Pasture/Hay, Cultivated Crops, Woody Wetlands, and Emergent Herbaceous Wetlands.

Section S1.3 TempCNN training data

Neural networks benefit from large, representative datasets to learn to classify input data to corresponding labels. We randomly sampled one hundred thousand pixels across Minnesota. At each sampled location, we extracted Landsat spectral data—spanning the periods of March/April, May/June, and July/August—across three consecutive years, along with the corresponding latitude, longitude, and land cover class for an NLCD year (Figure 1). For example, in the 2019 NLCD year, we obtained Landsat data from 2018, 2019, and 2020, along with the latitude, longitude, and land cover class from the NLCD map for one hundred thousand samples. We repeated this process for seven NLCD maps yielding approximately seven hundred thousand samples (Tables S1 & S2).

Section S1.4 Temporal convolutional neural network (TempCNN) architecture

We developed a supervised deep learning model (TempCNN) to classify leafy spurge and 15 additional land cover classes. The TempCNN architecture has shown to produce higher classification accuracy compared to shallower machine learning algorithms (e.g., Random Forests) for satellite image time series classification because it can directly learn from the temporal dimension of the data (Allred et al. 2021, Brock and Abdallah, 2022). The neural network contains a series of convolutional layers (1D convolutions), dropout layers, and fully connected layers.

In deep learning, convolutions are mathematical operations that are typically applied to images. Familiar examples of convolutions that are applied to images are "blur" or "sharpening" filters. Here, we use 1D convolutions to transform our inputs (a vector of spectral data from nine seasonal mosaics in our Landsat time-series) (i.e., March/April, May/June, and July/August for the three consecutive years) to learn the association between spectral and phenological signals and land cover classes. Specifically, a 1D convolution is a small filter that looks at a subset of the data and performs a mathematical transformation on the grouping of data to output new synthetic higher order information about the data group. The size of the convolution filter (i.e., the kernel size) can also vary to allow the model to learn from different sets of spectral data and wider phenological windows. The convolution then steps (or, slides) over the data to perform the same operation on the next group. The deep learning algorithm uses training to learn parameter values for the transformations of the convolutions that lead to the most accurate classification.

Deep learning models are subject to overfitting because of the large number of parameters. We used dropout layers to minimize the problem of overfitting. These layers randomly set a selection of parameters in the model to zero as a form of regularization to prevent the model from memorizing the training data. By randomly deactivating certain parameters during the training process, dropout encourages the network to learn in a more robust and generalizable manner.

Fully connected layers connect all parameters from previous layers to allow for learning between layers. Additionally, we included a concatenate layer to combine spectral and phenological features with location data (i.e., latitude, longitude) to provide spatial context during training. Overall, the model outputs a vector of probabilities for each land class for each pixel (range: 0-1). Finally, to generate pixel classifications to test model accuracy, we used an Argmax layer that selected the land cover class with the highest probability as the land class assignment. Additional details on the model architecture and supporting code are archived in Zenodo at https://doi.org/10.5281/zenodo.20315228.

Section S1.5. TempCNN model training

We randomly divided the approximately seven hundred thousand samples into an 80/10/10 ratio of training, validation, and testing data (Table S2). Training data is used as direct input for model training. The validation data is used to evaluate and fine-tune the model's parameters during training. The testing data is withheld from model training and used as an independent set to evaluate performance.

Training the deep learning model comprises two main steps - the forward pass and the backward pass.

Initially, the model's parameters (i.e., weights and biases) were randomly assigned from a uniform distribution. During the forward pass, samples of the input data (in this case, 8,192 samples of spectral data from our Landsat time-series) are fed through the model. As the data moves through each layer of the network, various computations are performed through a series of convolutions and dropout layers using the model's current parameters. The goal is to produce an output that is as close as possible to the actual

target output (the land cover class). The discrepancy between the predicted output and the actual land cover class is calculated as the training loss using a loss function. We used the categorical cross-entropy loss to measure the difference between predicted labels and true labels for each class, as is common in multi-class classification problems.

Once the loss is computed from the training data, the optimizer determines how to adjust the model's parameters to improve its performance. This is done during the backwards pass. During this stage, the model calculates gradients, which are essentially directions and magnitudes of change needed for each parameter to minimize the loss. The Adam optimizer (Kingma & Ba, 2014), a variant of stochastic gradient descent, uses these gradients to update the model's parameters. Adam is popular because it incorporates adaptive learning rates and momentum, making the optimization process more efficient by speeding up convergence and stabilizing updates.

By repeating the forward and backward passes over multiple epochs—complete passes over the training dataset—the model progressively learns the intricate patterns in the data, allowing it to make accurate predictions when presented with new, unseen testing data. Throughout the process, the validation dataset helps in tuning model parameters to prevent overfitting. After each epoch, the loss is also computed against the entire validation dataset (i.e., the validation loss). Validation loss is crucial because it helps assess how well the model generalizes to new, unseen data. Ideally, the validation loss should also decrease as training progresses. However, if it starts to increase significantly while the training loss continues to decrease, this indicates that the model might be overfitting to the training data. As such, we trained the TempCNN for 100 epochs and monitored both the training and validation loss to prevent overfitting (Figure S1).

Due to the uneven number of samples per land cover class, we computed a class weight vector as the inverse frequency of class counts in the training dataset. Class weights were applied to the loss function during the training process to increase the importance of errors made on rare classes relative to more common classes (Cabezas et al., 2020) (Table S2).

We normalized all Landsat spectral bands prior to model training to ensure consistent feature scaling across inputs. We set the dropout rate at 10% (Brock & Abdallah, 2022). For convolutional layers, we used a kernel size of 3 and increased the number of kernels from 64 to 128 after the first layer. We used dilated convolutions with dilation rates of 1, 2, and 4 for each successive layer (Pelletier et al., 2019).

We trained the TempCNN using an adaptive learning rate for the Adam optimizer. Specifically, we used learning rate warmup and cosine decay. Initially set at 1e-7, the learning rate linearly increased to 1e-2 over the first 10 epochs, after which it gradually decayed in a cosine pattern for the remaining epochs. This strategy promotes stable initial training with a slow learning rate and efficient convergence to improve performance and generalization. We trained the TempCNN using Python v.3.7.1 with Tensorflow v.2.0.0 (Abadi et al., 2016) and Keras v.2.10.0 (Chollet, 2017).

Section S1.6 TempCNN model evaluation

We assessed model performance for each class for several evaluation metrics that are based on a class-wise confusion matrix from the Argmax output. The confusion matrix contains the number of correctly predicted true positives (TP), incorrectly predicted false positives (FP), correctly predicted true negatives (TN), and incorrectly predicted false negatives (FN) for all records in the withheld testing dataset.

The evaluation metrics we calculated were: Accuracy, Specificity, Sensitivity, Precision, and F2.

We calculated overall accuracy as the proportion of correctly identified pixels:

Accuracy = (TP + TN) / (TP + TN + FP + FN).

We calculated class-wise specificity, or the ability to discriminate true negatives (i.e. True Negative Rate (TNR)).

TNR = TN / (TN + FP).

We calculated class-wise sensitivity (also termed recall), or the ability to predict all true positives (i.e. True Positive Rate (TPR)).

TPR = TP / (TP + FN).

We calculated the F2-score per class. F2 is derived from precision (i.e. the proportion of all identified positives that are correct) and sensitivity (i.e. TPR, see above). F-scores are employed when both precision and sensitivity are important factors for model evaluation. F2 places more importance on sensitivity relative to precision. This is preferred as a performance metric for invasive species because the metric weighs false negatives greater than false positives due to the fact that the potential economic cost of falsely declaring an invasive species absent is high.

Precision = TP / (TP + FP).

F2-score = [(5 * precision * recall) / (4 * precision + recall)].

Section S1.7 TempCNN model predictions

We predicted the probability of leafy spurge and 15 additional land cover classes at a 30-meter resolution across Minnesota, totaling approximately 250 million pixels. The probability of each land cover class can be interpreted as the likelihood that the class is present in each pixel. As the TempCNN required the same data structure for prediction as used for training (i.e., three consecutive years of Landsat data), we predicted each land cover class across seven time-steps: 2000-2002, 2003-2005, 2006-2008, 2009-2011, 2012-2014, 2015-2017, and 2018-2020.

To gauge the predictive capabilities of our TempCNN across land cover classes, we evaluated the model against the NLCD's 2021 dataset. For each land cover class, we randomly sampled 10,000 points from the NLCD 2021 layer, extracted the predicted probability values at these locations from our most recent TempCNN model predictions (2018-2020), and plotted histograms to visualize agreement between our predictions and the NLCD dataset (Figure S9).

To further gauge the predictive capabilities of our TempCNN for leafy spurge, we evaluated performance on an independent set of occurrence points (N = 1,018) and polygons (N = 940) with reported population area estimates ('Infested Area, m^2') collected from iNaturalist and EddMaps between the years 2021-2023 (Figure S2).

We also examined how well the latest leafy spurge prediction (2018-2020) captures newly reported occurrences in 2021-2023. For each occurrence in 2021-2023, we extracted the predicted probability from the 2018-2020 leafy spurge model prediction and plotted these probabilities against the number of occurrence records (Figure S9).

To assess the models' ability to detect populations of different sizes, we used polygons with reported population area estimates (measured as 'Infested Area, m^2'). Because the polygons varied in size, we averaged predicted probabilities across all pixels intersecting each polygon. This approach ensured that we captured the variation in density and spatial heterogeneity within populations. We then fit quantile regressions between the mean predicted probability of leafy spurge and population area for a range quantiles to determine how population size influences the likelihood of leafy spurge occurrence using the 'rq' function from the 'quantreg' package (Koenker et al., 2018). To explore how the intercepts and slopes changed across all quantiles, we first fit the model with tau = -1, which explores quantiles (0,1). We then fit a representative sample to report with tau = c(0.05, 0.25, 0.5, 0.75) (tau = 0.5 represents regression on the median). This allowed us to examine whether the 2018-2020 model was more or less sensitive to detecting smaller infestations relative to larger ones (Figure 2).

Section S1.8 Spatial overlap and autocorrelation of occurrence datasets

We measured spatial autocorrelation for the community science occurrence and remotely-sensed prediction datasets independently (N = 1,771; remotely-sensed prediction dataset description below, see *Occurrence weighting for SDMs*; Figure 5). We used Moran's I to determine if occurrences were more clustered than random expectation. We calculated Moran's I using the 'moran.mc' function in the 'spdep' R package v1.3-4 (Bivand et al., 2015). We determined if the value was significantly greater than random at a significance level of 0.05 using permutation tests (n = 999).

To quantify the extent to which the community science and remotely-sensed predictions co-localized, we quantified spatial overlap between occurrences from the two datasets using the Jaccard index [range: 0 (no overlap) to 1 (complete overlap)]. We used a 0.1-degree latitude/longitude grid to create binary presence-absence maps for both datasets. We then calculated the Jaccard index as the intersection over union between both raster datasets using the 'raster' R package (Hijmans et al., 2015).

Section S1.9 Assessing spatial bias near roadsides

We measured the distance to primary and secondary roads for both community science occurrences and remotely-sensed prediction points using the U.S. Census Bureau's MAF/TIGER Database shapefiles for roads in Minnesota. We used the same occurrence points from the SDM analyses (N = 1,771 occurrences per dataset). We then used a k-d tree nearest neighbor search to calculate the shortest distance between occurrence points and road segments. For comparison, 1,771 random points were sampled from within the Minnesota state boundary and distances to roads were similarly calculated. We repeated the random point sampling process for 999 permutations and calculated the mean distance to roads. We evaluated spatial bias by comparing the mean distance of community science, remote sensing, and random observations to roadsides (Figure 5).

Section S1.10 Species Distribution Models

Section S1.10.1 Environmental Data

We downloaded three bioclimatic variables at a 30 arcsecond resolution (~1km) from WorldClim v.2.1 (worldclim.org): mean temperature of the warmest quarter (Bio 10), mean temperature of the coldest quarter (Bio 11), and annual precipitation (Bio 12) (Figure S4). These axes of climate variation are relevant to key life history stages of leafy spurge in Minnesota (Briscoe Runquist & Moeller, 2024; Lake et al., 2023). Warm season temperatures affect seed germination, growth, and phenology (Wolkovich et

al., 2013), cold season temperatures influence winter survival and damage (Chapman et al., 2017), and precipitation influences growth and reproduction (Petitpierre et al., 2017).

Section S1.10.2 Occurrence sampling for SDMs

We fit SDMs using both community science occurrences and remotely-sensed predictions (R Core Team. 2023). For SDM development, the starting high-confidence remotely-sensed predictions dataset combined the continuous and new early and late occurrence points (see *Geography and timing of new occurrences and extirpations*). The community science and remotely-sensed datasets were thinned using grid sampling to match the raster resolution of the environmental data. After thinning, 1,771 and 22,204 occurrence records remained, respectively. To obtain remotely-sensed occurrence data for SDM development, we created 10 subsamples of 1,771 points from the 22,204 remotely-sensed occurrences. To generate these subsamples, we first upsampled the points using a spatial grid by a factor of 4 (new resolution of ~ 2 arc minutes) to preserve the spatial information of the TempCNN prediction using the gridSample function in dismo package. gridSample allows for upsampling in a checkerboard to help lessen spatial autocorrelation, so half of the subsamples were created using 'white' fields and half using 'black' fields. We then randomly sampled 1,771 occurrences from each of the ten upsampled datasets to generate the final remotely-sensed subsampled datasets.

Section S1.10.3 Occurrence weighting for process-based models

We developed unweighted (traditional) and weighted (process-based) SDMs to evaluate the extent to which remotely-sensed demographic information improved the accuracy of SDMs. We weighted occurrences by: (1) leafy spurge softmax prediction from the TempCNN and (2) the slope from linear regressions of the softmax probability for leafy spurge on time (population trend).

Section S1.10.4 Modeling algorithms

We used the ENMeval package to generate a collection of nine Maxent models that reflected a set of commonly used hyperparameter combinations for each modeled dataset. ENMeval models were run separately for each occurrence dataset and weighting combination, resulting in 4 general model types that were represented by a population of individual models with different hyper parameter combinations (community science (traditional), remote sensing (traditional), remote sensing (probability-weighted), remote sensing (slope-weighted). For the community science dataset, we used traditional presence/background models with each of the hyperparameter combinations to generate nine community science SDMs. For the remote sensing models, there were 10 subsampled datasets to assess variability generated due to subsampling error. Therefore, each remote sensing model type (traditional, probability-weighted, slope-weighted) had 90 SDMs (9 hyperparameter combinations x 10 data subsets).

Assessing multiple models in each group allowed us to evaluate model stability, model discrimination, and the potential for overfit (Radosavljevic & Anderson, 2014). ENMeval allows the user to vary the feature classes used (linear (L), quadratic (Q), product (P), threshold (T) and hinge (H)) and the smoothing regularization parameter (beta). We developed models using three feature class combinations that vary in complexity (LQH, LQHP, LQHPT) and three beta parameters (1, 2, 3).

To develop the Maxent models, we generated 10,000 random background points to characterize the distribution of environmental conditions across Minnesota. We used four-fold cross-validation by randomly partitioning occurrences and background points into spatial blocks using 'checkerboard 2'

partitioning to reduce spatial autocorrelation among cross-validation blocks (Figure S5). We then ran the ENMeval function using the 'maxnet' implementation of the maximum entropy model.

To weight occurrences in Maxent models, we generated "pseudopresences" for models using probability quantiles from data output from our remote sensing model. The "pseudopresences" were records added to the dataset that replicated the environmental values of occurrences where the number of replicates of an occurrence indicated the weighting (Eckhart et al., 2011). For weighting by the remote sensing model's probability estimates, occurrence records with a probability below the 25th percentile were represented once in the dataset, records between the 25th and 50th were represented twice, records between 50th and 75th were represented three times, records between the 75th and 99th were represented four times, and those above the 99th percentile were represented five times. To generate weighting for demographic growth estimates (slope-weighted), we repeated this process but used slopes to generate the quantile distribution.

For BRT models, we also generated a collection of nine models for each occurrence dataset and weighting combination. This allowed us to represent a set of commonly used hyperparameters and to assess model stability, discrimination and overfit. We selected BRT hyperparameters for each set of models based on practices outlined in Elith et al. (Elith et al., 2008). We varied tree complexity (2, 3, or 5) and learning rate (three values between 0.001 and 0.01). The algorithm required that weighting values be positive, so in population size change models, we assigned randomly generated very small positive values (mean: 1e-4) for records with negative slopes (RS: N = 74). During model training we set the bag fraction to 0.8. Learning rates varied slightly to ensure that we produced well-regularized models that reach an asymptote in model deviance within 10,000 trees.

To develop the BRT models, we generated absence points equal to the number of presences. For community science models, we sampled pseudoabsences from across the sample area as is common practice in SDM studies (Elith et al., 2010). For the remotely-sensed dataset, we sampled absence points from regions where the remote sensing models predicted leafy spurge at less than five percent probability (Figure S6). We built models using the 'gbm.step' function from the 'dismo' and 'gbm' packages to select the model with the optimized deviance for the hyperparameters chosen (Greenwell et al., 2019; Hijmans et al., 2017).

Section S1.10.5 Model evaluation metrics

We evaluated SDM performance using multiple metrics. We used the average Area Under the Curve (AUC) across four cross-validation folds to assess model discrimination (Kass et al., 2021; Radosavljevic & Anderson, 2014). AUC measures how well the model distinguishes between presence and absence, with values ranging from 0.5 (random) to 1.0 (excellent). To detect model overfitting, we calculated the average difference between the calibration and evaluation AUC values across the four cross-validation folds. The AUC difference quantifies the degree to which the model's predictive performance on the training data (calibration) exceeds its performance on unseen data (evaluation), where higher values indicate a greater extent of overfitting (Radosavljevic and Anderson 2014).

AUC can be misleading due to its equal weighting of omission and commission errors, which may be especially problematic for invasive species where omission errors may be more costly (Leroy et al., 2018; Lobo et al., 2008). Therefore, we also calculated sensitivity, which measures the proportion of correctly predicted occurrences. We calculated sensitivity using the 1,018 occurrence records collected from iNaturalist and EddMaps between 2021-2023 (Figure S2). These records were not used during model training and represent an independent dataset composed of the type of data (e.g. new occurrence records)

that SDMs are often developed to predict. Sensitivity was evaluated at a threshold that maximized the difference between the true positive rate and the true negative rate. One of the potential strengths of remote sensing is in detecting occurrences in difficult-to-survey, remote regions. To evaluate model sensitivity in rural compared to urban areas, we divided our data between the Twin Cities Metropolitan region and outlying rural areas (Figure S7).

We tested for significant differences in AUC, overfitting (AUC difference), and sensitivity among our four model types (community science, remote sensing (traditional, probability-weighted, slope-weighted)) using linear mixed-effect models. For each evaluation metric, we included model type as a fixed effect and dataset (e.g. remotely-sensed subsample dataset id) as a random effect. We checked models to ensure that they conformed to assumptions of normality and homogeneity of variance in residuals. We assessed significance using the 'Anova' function from the 'car' package in R using type II sums of squares (Fox & Weisberg, 2018). We performed Tukey's tests to evaluate the significance of pairwise comparisons using the 'emmeans' R package (Lenth, 2025).

Section S2: Results

Section S2.1 Spatial biases in occurrences

The spatial overlap between remotely-sensed and community science occurrences was modest (Jaccard's Index = 0.36) (Figure 5). Spatial autocorrelation was significant and similar for the community science occurrences (Moran's I = 0.221; $p < 0.001$) and remotely-sensed predictions (Moran's I = 0.242; $p < 0.001$). These results indicate that occurrences tend to be locally clustered in both datasets.

Section S2.2 Boosted Regression Tree (BRT) results

Similar to Maxent results, model type had a large effect on BRT evaluation metrics and suitability predictions (Figures S10 & S11). Discrimination was higher for remotely-sensed SDMs (AUC: CS: 0.915 ± 0.005; RS: unweighted: 0.970 ± 0.001, prob-weighted: 0.945 ± 0.001, slope-weighted: 0.942 ± 0.001) and overfit was higher for community science SDMs (AUC difference: CS: 0.078 ± 0.001; RS: unweighted: 0.026 ± 0.0004, probability-weighted: 0.013 ± 0.0004, slope-weighted: 0.012 ± 0.0004). Community science and Remotely-sensed models had similar overall sensitivity for independent testing data (Sensitivity: CS: 0.81 ± 0.03; RS: unweighted: 0.82 ± 0.01, probability-weighted: 0.81 ± 0.01, slope-weighted: 0.80 ± 0.01) and similar to Maxent SDMs, BRT models had much better sensitivity in non-urban settings (Non-urban sensitivity: CS: 0.58 ± 0.02; RS: unweighted: 0.74 ± 0.005, probability-weighted: 0.75 ± 0.005, slope-weighted: 0.75 ± 0.005) (Figure S10). Again, weighting made little difference in evaluation metrics (Figure S10). Model projections using remotely-sensed SDMs indicated a much wider and more contiguous area of high habitat suitability. This was reflected in the greater regularization of the response curves for environmental variables (Figure S11).

Section S3: Supplementary Figures

Figure S1. Training history of the temporal convolutional neural network (TempCNN).

The left panel shows the training (blue) and validation (orange) loss across 100 epochs, while the right panel displays training and validation accuracy over the same period. The training loss and accuracy steadily improve and converge.

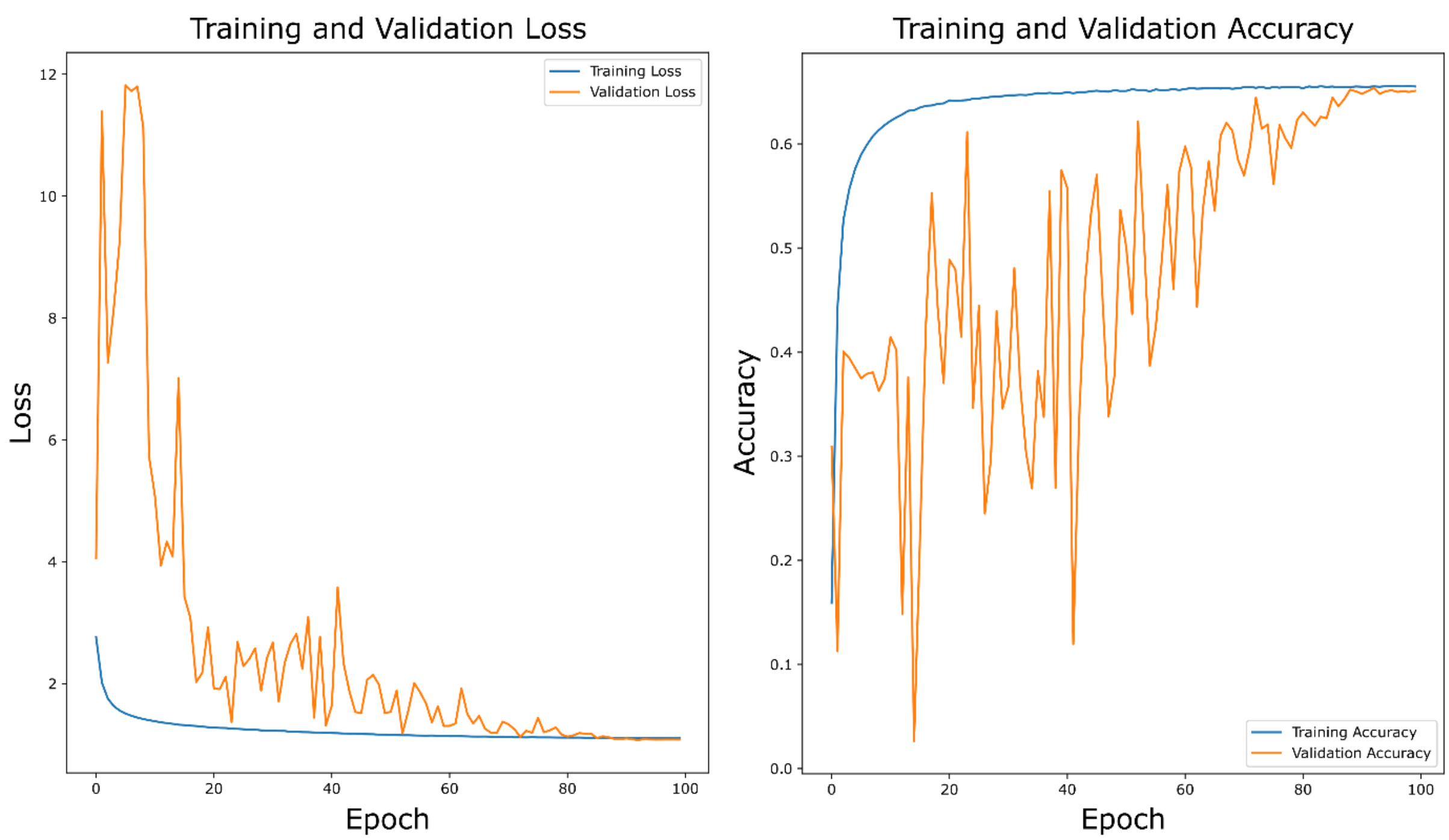

Figure S2. Occurrence points and polygon records of leafy spurge in Minnesota.

The left panel shows occurrence points (blue) from 2000-2020 (N=3,345). The middle panel shows occurrence points (red) from 2021-2023 used for TempCNN model evaluation (N=1,018). The right panel shows polygon centroids (green) with reported population size estimates from 2021-2023 used to evaluate the TempCNN model (N=940).

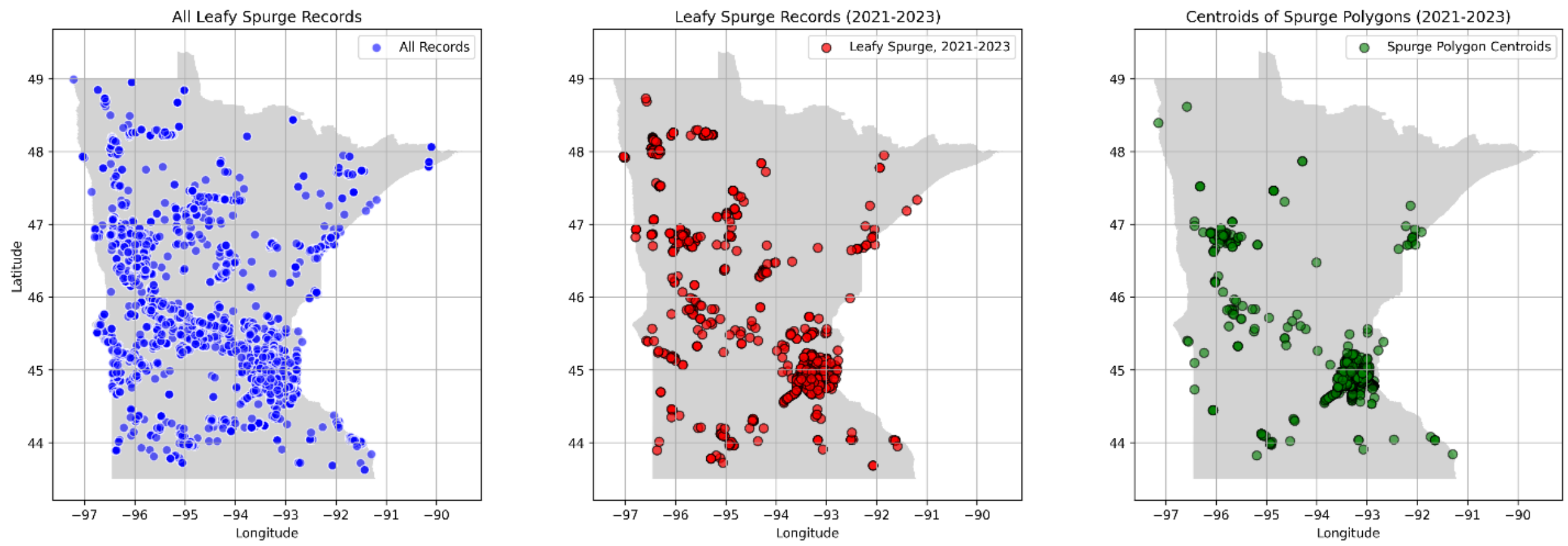

Figure S3. Sensitivities of CS occurrences.

We transformed TempCNN predictions of leafy spurge into discrete predicted occurrence points. We chose a probability threshold for a positive leafy spurge presence of 0.5. We chose this threshold because it indicated when the probability of leafy spurge was greater than the sum of the probability of all other classes. It may be taken as a conservative threshold estimate because it was greater than the threshold required to obtain 75% sensitivity of occurrences reported by community scientists using the 2009-2011 prediction period as a midpoint (threshold = 0.38 estimated at the 2009-2011 midpoint prediction period).

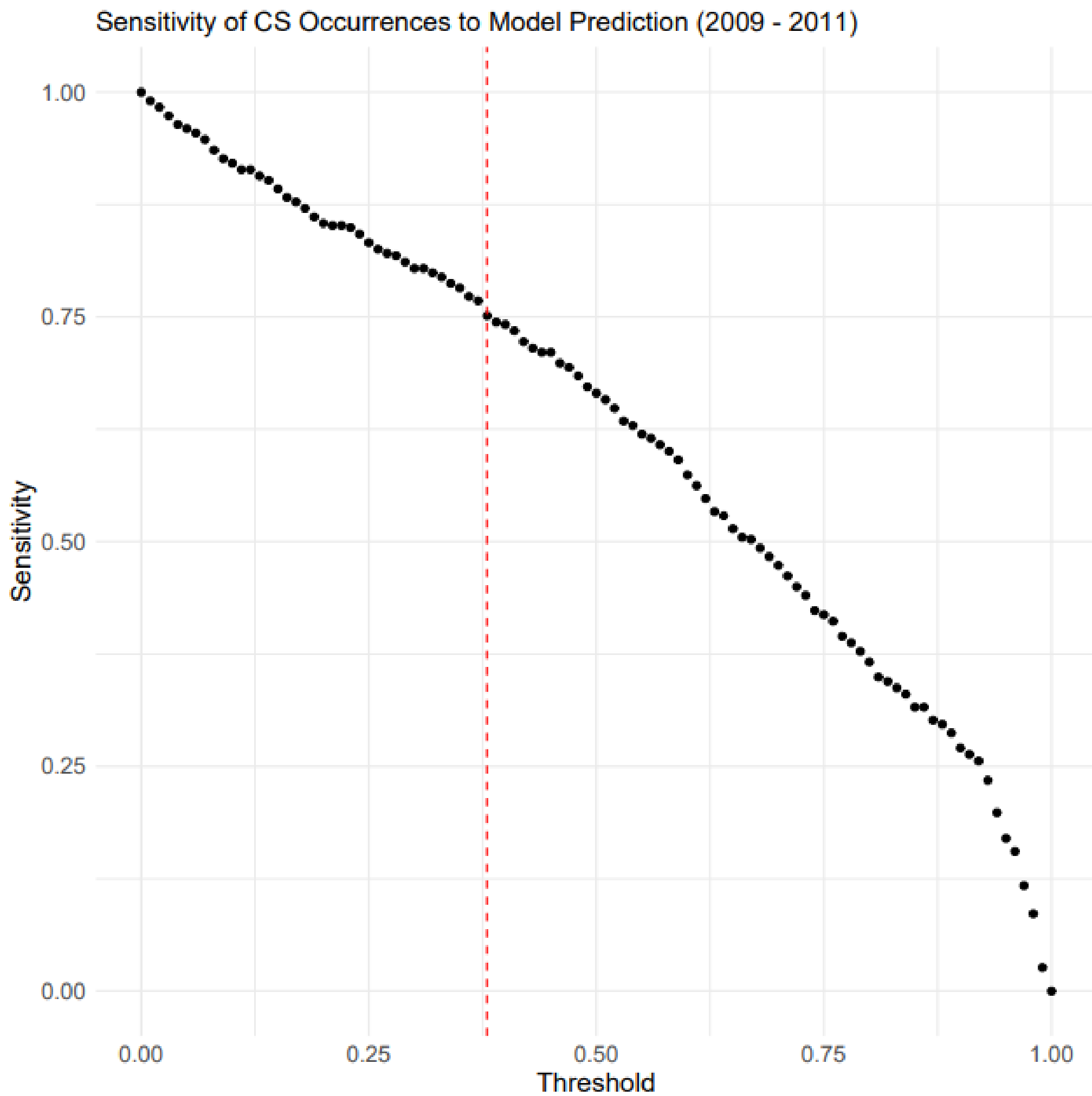

Figure S4. Bioclimatic variables used for species distribution modelling.

Mean temperature of the warmest quarter (Bio 10), mean temperature of the coldest quarter (Bio 11), and annual precipitation (Bio 12).

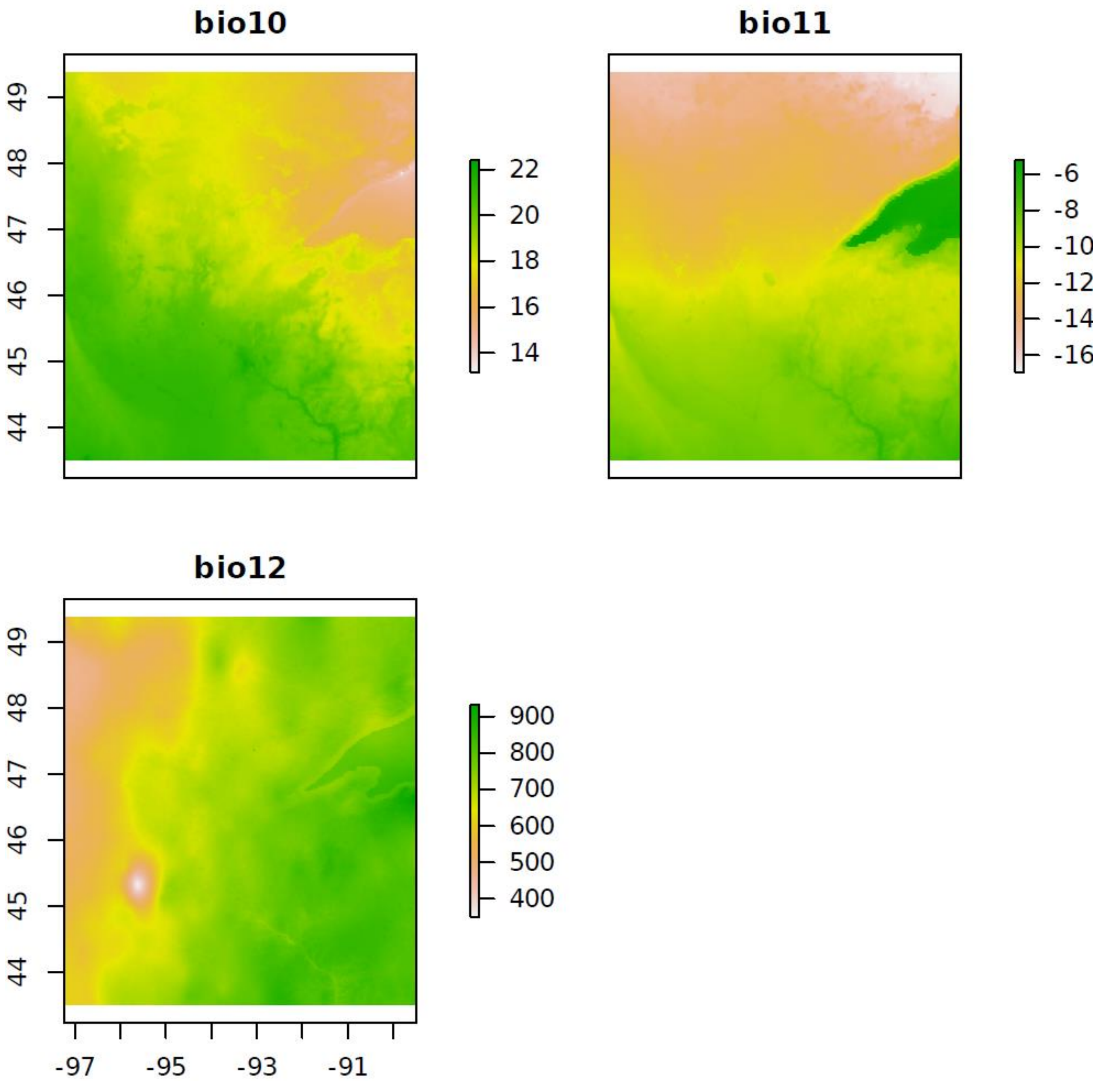

Figure S5. Cross-validation spatial blocks for species distribution models using community science and remote sensing occurrences datasets.

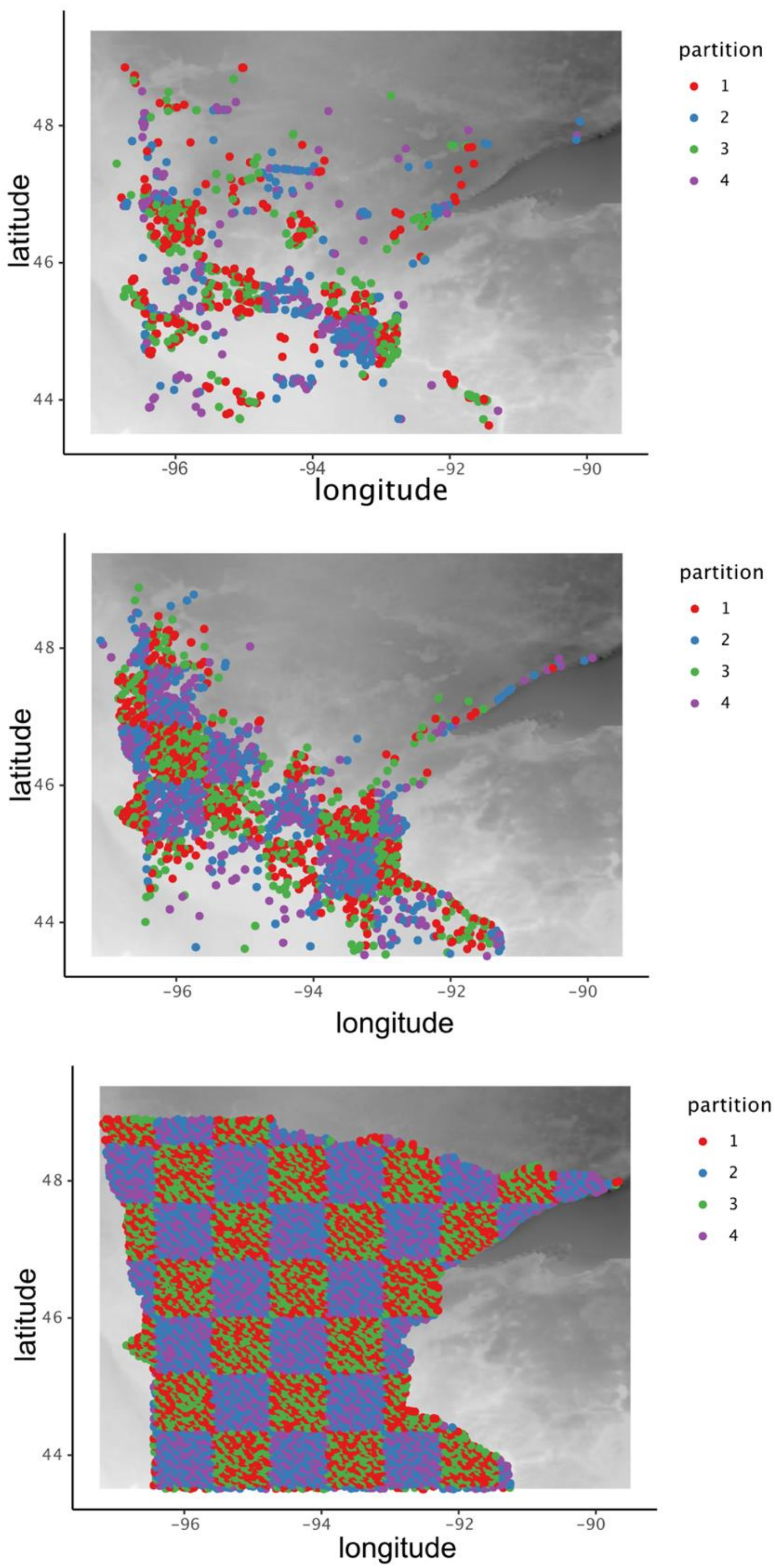

Figure S6. Binary map of the predicted probability of leafy spurge.

Areas with < 0.05 probability shown in green, and areas > 0.05 probability shown in white. Absence points were sampled in areas with a < 0.05 probability for the Boosted Regression Tree (BRT) models.

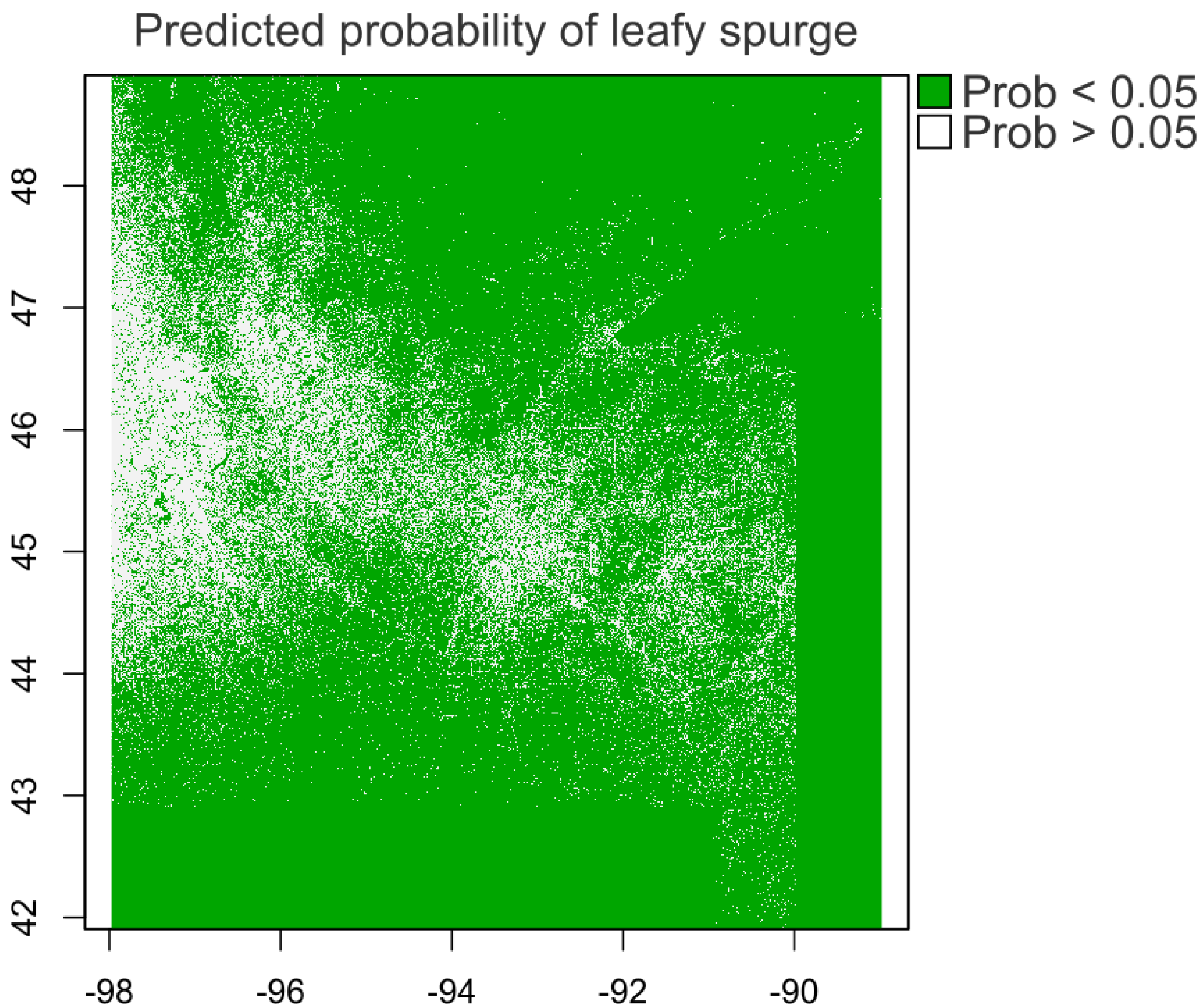

Figure S7. Independent records of leafy spurge from 2021-2023 partitioned by geographic region.

Red squares indicate records sampled from the surrounding metropolitan region of Minnesota (including the cities of Minneapolis and St. Paul, MN). Blue circles indicate records sampled outside of the metropolitan center. When evaluating SDMs, we calculated sensitivity separately for all points and non-metropolitan points (blue) to assess model discrimination in different geographic contexts.

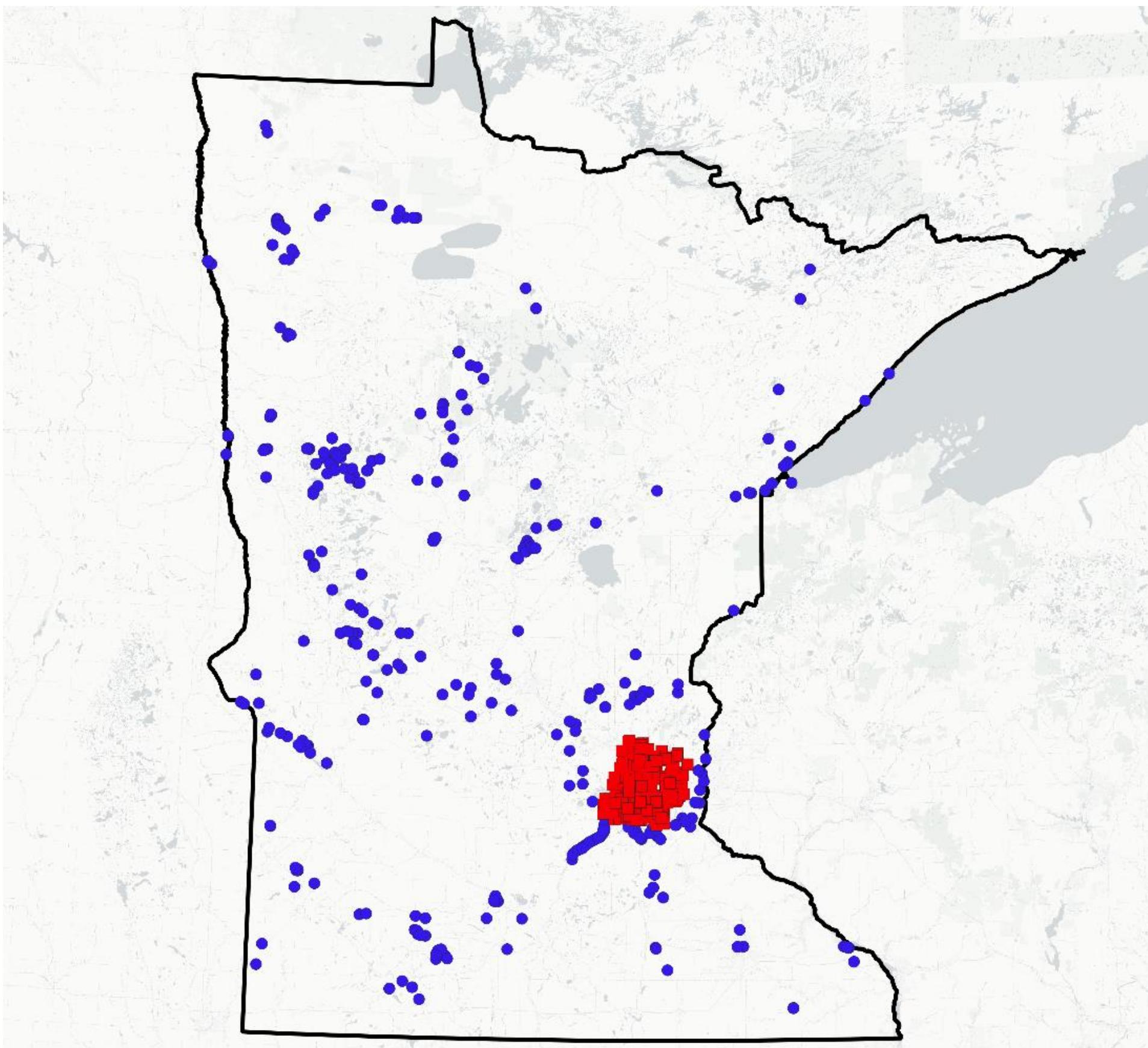

Figure S8. Land Classification Masks with Confusion Matrix of True Positive Rates

(A-B) Land classification prediction for (A) 2001 and (B) 2019 time period predicted by the TempCNN (C) Matrix shows the classification results of the temporal convolutional neural network based on the 10% withheld testing dataset. Classification results were calculated from the testing dataset for 15 land cover classes and leafy spurge. Values in the confusion matrix are normalized along rows, where the diagonal represents the proportion of records for which the predicted class is equal to the true class (i.e., true positive rate). Off-diagonal elements are misclassifications between the true class and the predicted class.

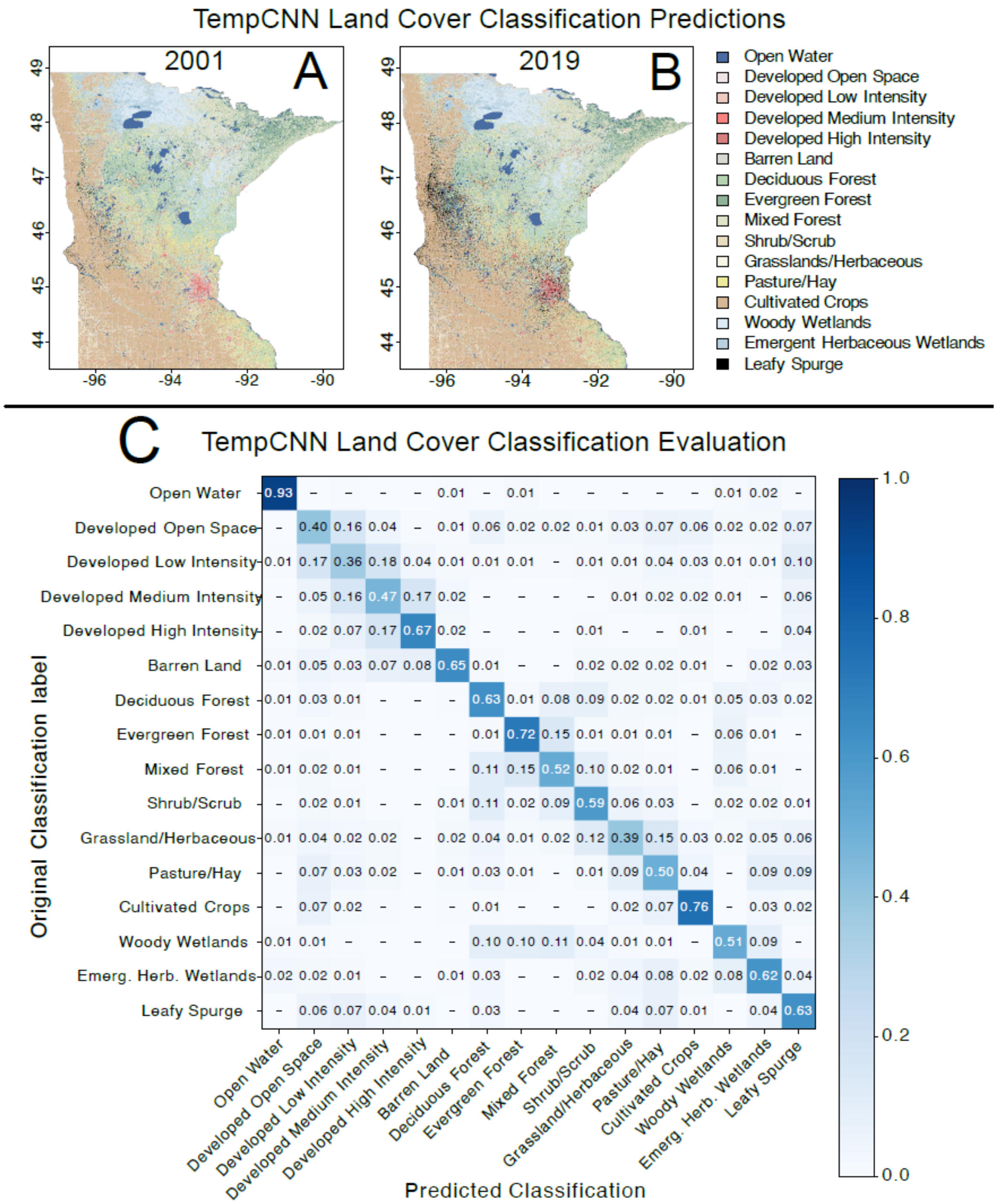

Figure S9. Comparison of predicted land cover class probabilities from our latest prediction (2018-2020) with the National Land Cover Database's 2021 dataset.

Histograms of predicted probabilities for 15 predicted land cover classes (purple histograms) were created by randomly sampling 10,000 points per land cover class from the NLCD 2021 dataset and extracting the probability values for these points from our latest predictions. In addition, we generated a leafy spurge prediction across Minnesota using Landsat imagery from 2018-2020 and determined if recently reported occurrences (from 2021-2023, N = 1,018) were identified with high probability by plotting the histogram of probabilities (red histogram). For each plot, the Y-axis indicates the number of random land cover class samples. The X-axis indicates the probability of each land cover class predicted in 2018-2020. The dashed vertical line indicates the median probability for each histogram.

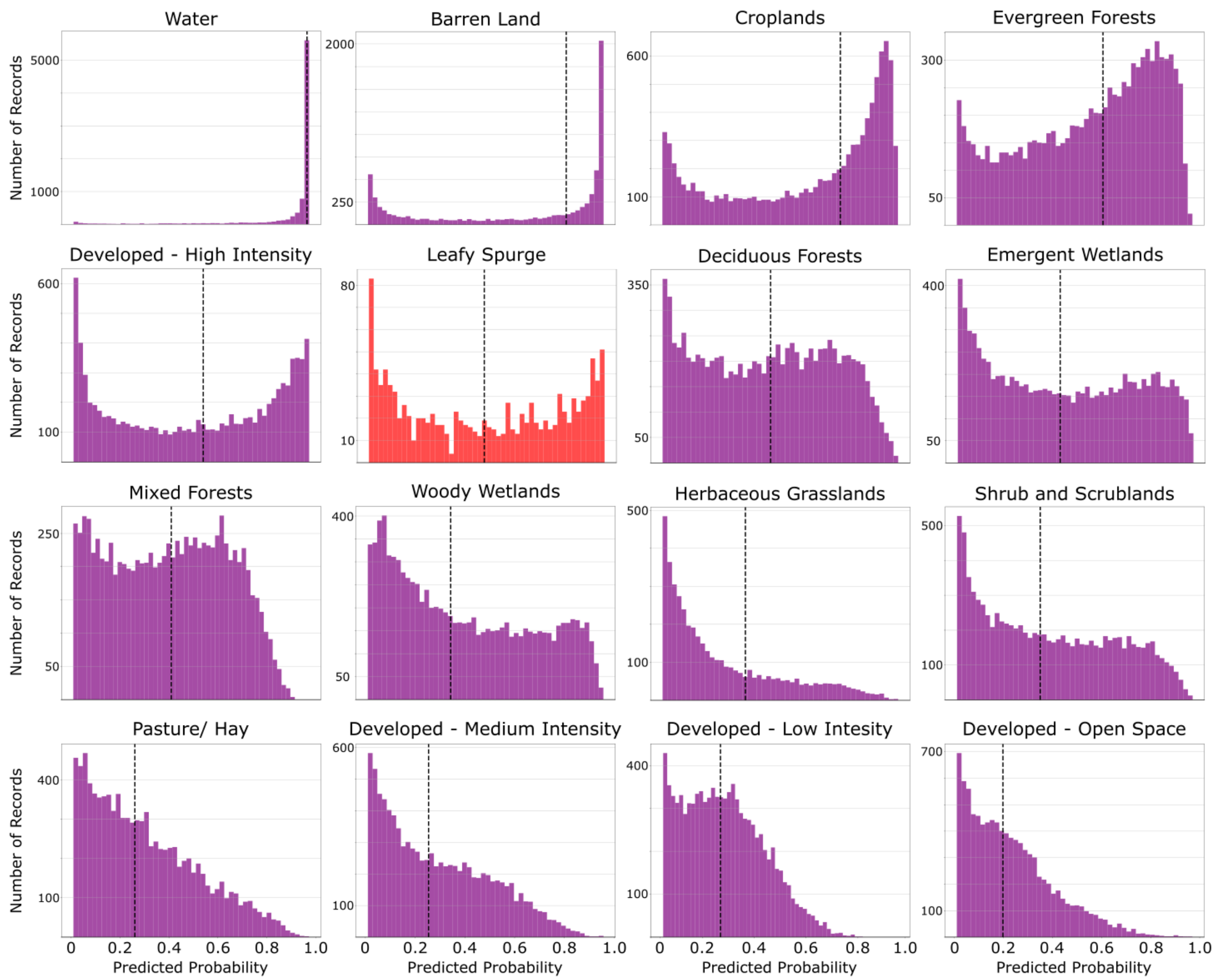

Figure S10. Comparison of species distribution model evaluation metrics.

Maxent and Boosted Regression Tree (BRT) models were developed using either community science or remotely-sensed occurrences. There were four model types based on the occurrence datasets used to build models: 1) unweighted community science occurrences, 2) unweighted remotely-sensed occurrences, 3) probability-weighted remotely-sensed occurrences, and 4) slope-weighted remotely-sensed occurrences. Remote-sensed models were built for 10 subsamples of the larger population of predicted leafy spurge occurrences. Each model type is represented by a population of models that were built using nine different combinations of hyperparameter values (maxent: three features combinations and three regularization betas; BRT: three tree complexity values and three learning rates); point colors represent model feature combination complexity (Maxent) and model complexity (BRT). Purple points are from models with less complexity and cyan points are from models with greater complexity (A & E) Model discrimination is the average AUC across four cross-validation folds, where higher values indicate higher discrimination ability. (B & F) Model overfit measures the average difference between the calibration and evaluation AUC values, with higher values indicating greater overfit. (C & G) Sensitivity to all independent leafy spurge records sampled from 2021 – 2023, where higher values indicate higher performance. (D & H) Sensitivity to independent records sampled from 2021 – 2023 from non-urban areas, where higher values indicate higher performance.

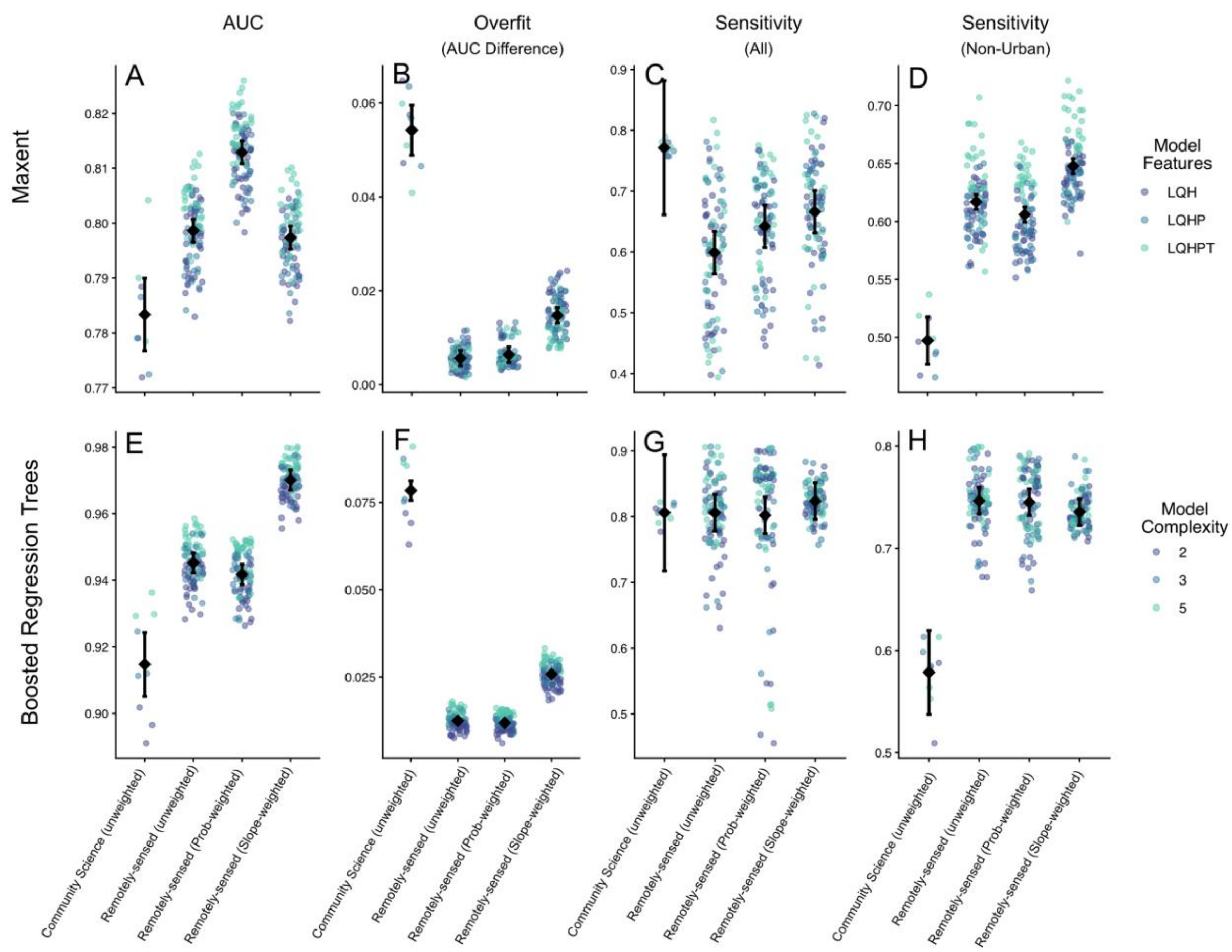

Figure S11. Boosted regression tree model predictions of climate suitability and partial response curves

(A, C, E, G) Boosted regression tree model projections of climate suitability from models built using either community science occurrences (unweighted) or remotely-sensed predictions (unweighted, probability-weighted, and slope-weighted). Each model projection is the average of models built with different combinations of hyperparameters and subsample datasets (n = 9 for community science, n = 90 for remotely-sensed). (B, D, F, H) Partial response curves for all models for the three environmental variables for each model type.

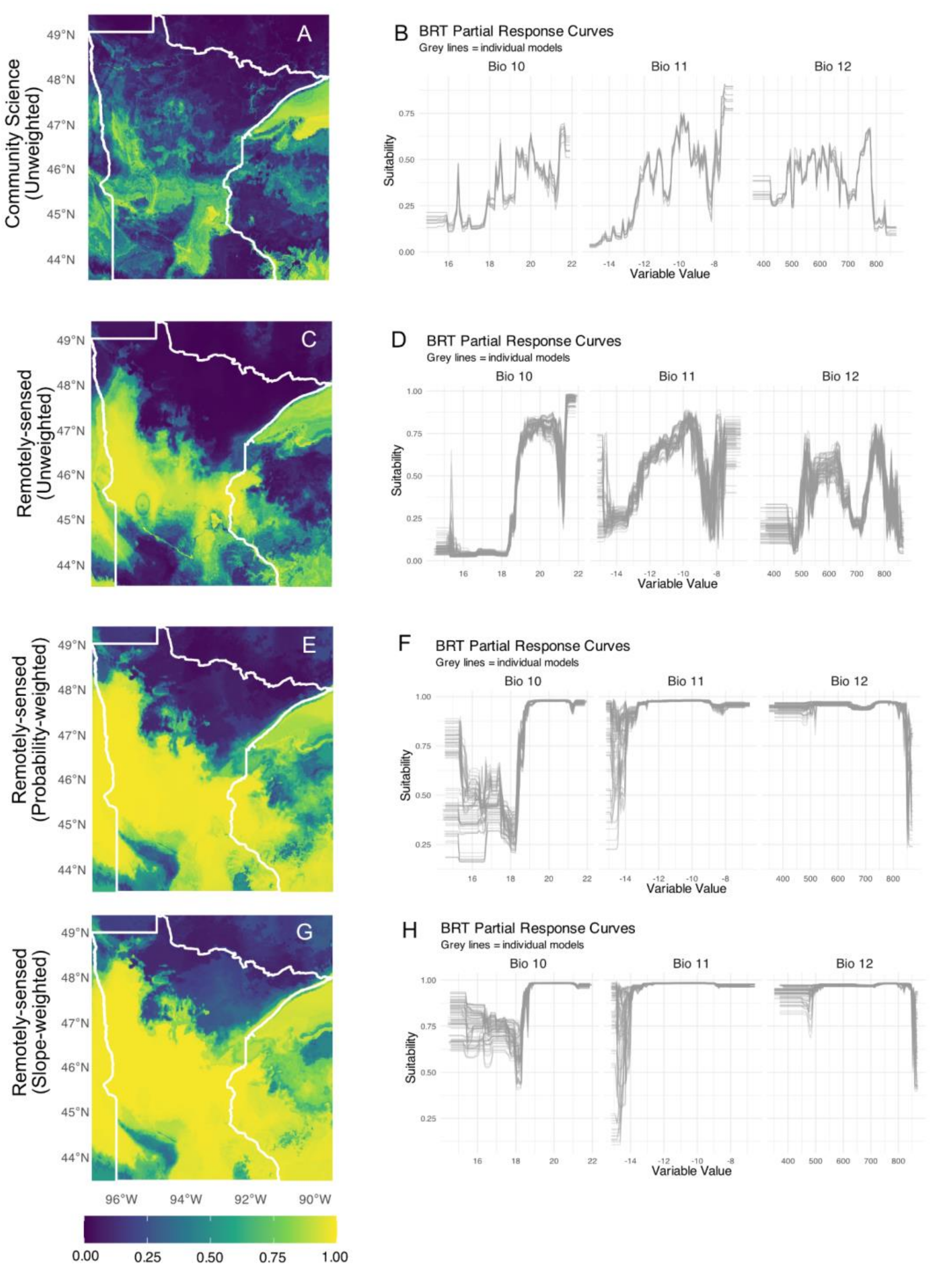

Section S4: Supplementary Tables

Table S1. Community science occurrence data of leafy spurge from 2000 to 2023.

Year	EddMaps	iNaturalist	Total	NLCD Year
2000	31	0	31	2001
2001	26	0	26	
2002	6	0	6	
2003	4	0	4	2004
2004	96	0	96	
2005	35	0	35	
2006	387	0	387	2008
2007	49	0	49	
2008	183	1	184	
2009	140	0	140	2011
2010	175	0	175	
2011	80	1	81	
2012	122	0	122	2013
2013	35	3	38	
2014	30	1	31	
2015	44	2	46	2016
2016	116	1	117	
2017	68	9	77	
2018	100	20	120	2019
2019	568	73	641	
2020	862	77	939	
2021	383	89	472	N/A
2022	315	98	413	
2023	73	60	133	
Total Leafy Spurge Occurrences (2000-2020)			3345	

Table S2. Number of training, validation, and testing records.

Numbers are Landsat satellite pixels for each land cover class and leafy spurge occurrences. Corresponding class weights calculated as the inverse proportion of the number of records in the training data for training the temporal convolutional neural network.

Class	Training Samples	Testing Samples	Validation Samples	Class weight
Cultivated Crops	214134	26734	26466	2.6
Woody Wetlands	86666	10820	10712	6.4
Deciduous Forest	62000	7741	7663	9.0
Emergent Herbaceous Wetlands	46052	5749	5691	12.1
Pasture/ Hay	32310	4033	3993	17.2
Open Water	30184	3768	3731	18.4
Mixed Forest	27185	3394	3360	20.4
Developed Open Space	14700	1835	1817	37.8
Evergreen Forest	12626	1576	1560	44.0
Developed Low Intensity	9142	1141	1130	60.8
Grassland/ Herbaceous	6794	848	840	81.8
Shrub/ Scrub	4583	572	566	121.3
Developed Medium Intensity	4422	552	547	125.7
Leafy Spurge	2680	335	330	207.4
Developed High Intensity	1403	175	173	396.2
Barren Land	1012	127	125	549.3

Table S3. Temporal convolutional neural network model performance and evaluation metrics. Model performance for leafy spurge and 15 additional land cover classes are based on the number of true positives (TP), false positives (FP), true negatives (TN) and false negatives (FN) in the testing dataset.

Class Name	TP	TN	FP	FN	Accuracy	Sensitivity	Specificity	Precision	F2
Leafy Spurge	217	67223	1842	118	0.97	0.65	0.97	0.11	0.32
Open Water	3506	65256	376	262	0.99	0.93	0.99	0.9	0.92
Developed Open Space	714	64477	3088	1121	0.94	0.39	0.95	0.19	0.32
Developed Low Intensity	414	67086	1173	727	0.97	0.36	0.98	0.26	0.34
Developed Medium Intensity	260	68282	566	292	0.99	0.47	0.99	0.31	0.43
Developed High Intensity	119	69010	215	56	0.99	0.68	0.99	0.36	0.58
Barren Land	89	68848	425	38	0.99	0.7	0.99	0.17	0.44
Deciduous Forest	4927	59499	2160	2814	0.93	0.64	0.96	0.7	0.65
Evergreen Forest	1136	66094	1730	440	0.97	0.72	0.97	0.4	0.62
Mixed Forest	1786	63872	2134	1608	0.95	0.53	0.97	0.46	0.51
Shrub/ Scrub	332	67208	1620	240	0.97	0.58	0.98	0.17	0.39
Grassland/ Herbaceous	331	66787	1765	517	0.97	0.39	0.97	0.16	0.3
Pasture/ Hay	1955	62555	2812	2078	0.93	0.48	0.96	0.41	0.47
Cultivated Crops	20135	42113	553	6599	0.90	0.75	0.99	0.97	0.79
Woody Wetlands	5580	57257	1323	5240	0.91	0.52	0.98	0.81	0.56
Emergent Herbaceous Wetlands	3547	61081	2570	2202	0.93	0.62	0.96	0.58	0.61

Table S4. Area of leafy spurge invasion in square kilometers predicted from the TempCNN model between 2000 and 2020.

Years	Invaded Area (sq. km)
2000 - 2002	1,067
2003 - 2005	2,417
2006 - 2008	4,149
2009 - 2011	3,124
2012 - 2014	1,372
2015 - 2017	2,134
2018 - 2020	7,156

Table S5. Sequential analysis of deviance table for Palmer Drought Severity Index.

Model tests are for quasipoisson generalized linear model testing for a relationship between the predicted extent of leafy spurge invasion with mean Palmer Drought Severity Index (PDSI) values and across different geographic regions. Significant P-values ($P < 0.05$) are shown in bold.

Source	df	X^2	F	Deviance	Resid. Deviance	P
NULL	62				44002571	
Mean PDSI	1	50.04	35.87	4496364	39506206	**< 0.001**
Region	8	266.93	33.37	33458118	6048088	**< 0.001**
Mean PDSI * Region	8	3.90	0.49	487446	5560642	0.867(X^2) / 0.860 (F)
Residual	45					

*Dispersion parameter = 125344

Table S6. ANOVA tables to test for difference in four SDM evaluation metrics.

Differences in evaluation metric values were tested (AUC, Overfit, Sensitivity (All), and Sensitivity (Non-urban)) for different model types based on the datasets used to build models: community science (unweighted), remotely sensed (unweighted), remotely-sensed (probability-weighted), and remotely-sensed (slope-weighted). The table presents results for two algorithms: A. Maxent (top) and B. Boosted Regression Trees (BRT; bottom). Significant P-values ($P < 0.05$) shown in pink.

A. Summary ANOVA Results for Linear Mixed-Effects Models of Maxent evaluation metrics

Model	Term	Sum Sq	Mean Sq	NumDF	DenDF	F value	Pr(>F)
Discrimination (AUC)	model_type	0.014	0.005	3	35.543	148.709	< 0.001
Overfit (AUC Difference)	model_type	0.010	0.003	3	34.060	330.960	< 0.001
Sensitivity	model_type	0.257	0.086	3	35.551	11.874	< 0.001
Sensitivity (Non-urban)	model_type	0.191	0.064	3	85.784	79.450	< 0.001

B. Summary ANOVA Results for Linear Mixed-Effects Models of BRT evaluation metrics

Model	Term	Sum Sq	Mean Sq	NumDF	DenDF	F value	Pr(>F)
Discrimination (AUC)	model_type	0.045	0.015	3	27.968	490.713	< 0.001
Overfit (AUC Difference)	model_type	0.029	0.010	3	55.662	1146.664	< 0.001
Sensitivity	model_type	0.024	0.008	3	53.339	1.750	0.168
Sensitivity (Non-urban)	model_type	0.054	0.018	3	30.298	34.438	< 0.001

References

Abadi, Martín, Paul Barham, Jianmin Chen, Zhifeng Chen, Andy Davis, Jeffrey Dean, Matthieu Devin, Sanjay Ghemawat, Geoffrey Irving, Michael Isard, et al. 2016. “TensorFlow: A System for Large-Scale Machine Learning.” *In Proceedings of the 12th USENIX Symposium on Operating Systems Design and Implementation* (OSDI 16), 265–283.

Allred, B. W., B. T. Bestelmeyer, C. S. Boyd, C. Brown, K. W. Davies, M. C. Duniway, L. M. Ellsworth, et al. 2021. "Improving Landsat Predictions of Rangeland Fractional Cover with Multitask Learning and Uncertainty." *Methods in Ecology and Evolution* 12(5): 841–849.

Bivand, M., R. Altman, L. Anselin, and R. Assunção. 2015. "Package spdep." *The Comprehensive R Archive Network*. https://cran.r-project.org/web/packages/spdep/index.html.

Briscoe Runquist, R. D., and D. A. Moeller. 2024. "Isolation by Environment and Its Consequences for Range Shifts with Global Change: Landscape Genomics of the Invasive Plant Common Tansy." *Molecular Ecology* 33(16): e17462.

Brock, J., and Z. S. Abdallah. 2022. “Investigating Temporal Convolutional Neural Networks for Satellite Image Time Series Classification.” *arXiv* [cs.CV]. http://arxiv.org/abs/2204.08461.

Cabezas, M., S. Kentsch, L. Tomhave, J. Gross, M. L. L. Caceres, and Y. Diez. 2020. “Detection of Invasive Species in Wetlands: Practical DL with Heavily Imbalanced Data.” *Remote Sensing* 12(20): 3431.

Chapman, D. S., R. Scalone, E. Štefanić, and J. M. Bullock. 2017. "Mechanistic Species Distribution Modeling Reveals a Niche Shift During Invasion." *Ecology* 98(6): 1671–1680.

Chollet, François. 2017. “Deep Learning with Python.” 1st ed. Manning Publications Co.

Eckhart, V. M., M. A. Geber, W. F. Morris, E. S. Fabio, P. Tiffin, and D. A. Moeller. 2011. "The Geography of Demography: Long-Term Demographic Studies and Species Distribution Models Reveal a Species Border Limited by Adaptation." *The American Naturalist* 178(S1): S26–S43.

Elith, J., J. R. Leathwick, and T. Hastie. 2008. "A Working Guide to Boosted Regression Trees." *Journal of Animal Ecology* 77(4): 802–813.

Elith, J., M. Kearney, and S. Phillips. 2010. "The Art of Modelling Range-Shifting Species." *Methods in Ecology and Evolution* 1(4): 330–342.

Fox, J., and S. Weisberg. 2018. "An R Companion to Applied Regression." 3rd ed. SAGE Publications.

Greenwell, B., B. Boehmke, J. Cunningham, G. B. M. Developers, and M. B. Greenwell. 2019. "Package 'gbm.'" R Package Version 2(5): ftp://r-project.org/pub/R/web/packages/gbm/gbm.pdf.

Hijmans, R. J., S. Phillips, J. Leathwick, J. Elith, and M. R. J. Hijmans. 2017. "Package 'dismo.'" *Circles* 9(1): 1–68.

Hijmans, R. J., J. Van Etten, J. Cheng, M. Mattiuzzi, M. Sumner, J. A. Greenberg, O. P. Lamigueiro, A. Bevan, E. B. Racine, A. Shortridge, et al. 2015. "Package 'raster.'" R Package 734: 473.

Kass, J. M., R. Muscarella, P. J. Galante, C. L. Bohl, G. E. Pinilla-Buitrago, R. A. Boria, M. Soley-Guardia, and R. P. Anderson. 2021. "ENMeval 2.0: Redesigned for Customizable and Reproducible Modeling of Species' Niches and Distributions." *Methods in Ecology and Evolution* 12(9): 1602–1608.

Kingma, D. P., and J. Ba. 2014. "Adam: A Method for Stochastic Optimization." arXiv [cs.LG]. http://arxiv.org/abs/1412.6980.

Koenker, Roger, Sanford Portnoy, Pin T. Ng, Achim Zeileis, Philippe Grosjean, and Brian D. Ripley. 2018. "Package 'quantreg.'" Reference manual, The Comprehensive R Archive Network. https://cran.r-project.org/web/packages/quantreg/quantreg.pdf.

Lake, T. A., R. D. Briscoe Runquist, L. E. Flagel, and D. A. Moeller. 2023. "Chronosequence of Invasion Reveals Minimal Losses of Population Genomic Diversity, Niche Expansion, and Trait Divergence in the Polyploid, Leafy Spurge." *Evolutionary Applications* 16(10): 1680–1696.

Lenth, R. V. 2025. "emmeans: Estimated Marginal Means, aka Least-Squares Means." https://rvlenth.github.io/emmeans/

Leroy, B., R. Delsol, B. Hugueny, C. N. Meynard, C. Barhoumi, M. Barbet-Massin, and C. Bellard. 2018. “Without Quality Presence-Absence Data, Discrimination Metrics Such as TSS Can Be Misleading Measures of Model Performance.” *Journal of Biogeography* 45(9): 1994–2002.

Lobo, J. M., A. Jiménez-Valverde, and R. Real. 2008. “AUC: A Misleading Measure of the Performance of Predictive Distribution Models.” *Global Ecology and Biogeography* 17(2): 145–151.

Pelletier, C., G. I. Webb, and F. Petitjean. 2019. “Temporal Convolutional Neural Network for the Classification of Satellite Image Time Series.” *Remote Sensing* 11(5): 523.

Petitpierre, B., O. Broennimann, C. Kueffer, C. Daehler, and A. Guisan. 2017. “Selecting Predictors to Maximize the Transferability of Species Distribution Models: Lessons from Cross-Continental Plant Invasions.” *Global Ecology and Biogeography* 26(3): 275–287.

Radosavljevic, A., and R. P. Anderson. 2014. “Making Better Maxent Models of Species Distributions: Complexity, Overfitting and Evaluation.” *Journal of Biogeography* 41(4): 629–643.

R Core Team. 2023. “R: A Language and Environment for Statistical Computing.” R Foundation for Statistical Computing, Vienna, Austria.

Wolkovich, E. M., T. J. Davies, H. Schaefer, E. E. Cleland, B. I. Cook, S. E. Travers, C. G. Willis, and C. C. Davis. 2013. “Temperature-Dependent Shifts in Phenology Contribute to the Success of Exotic Species with Climate Change.” *American Journal of Botany* 100(7): 1407–1421.
